# Developmental disruption of the mediodorsal thalamus alters prefrontal dopamine D2 receptor expression and prefrontal–accumbal neuronal recruitment during latent inhibition

**DOI:** 10.3389/fnbeh.2026.1998199

**Published:** 2026-10-29

**Authors:** Zakaria Ouhaz, Hanane Iben-Daoudi, Mouna Darfaoui, David S. Tait, Fatima-zahra Lamghari Moubarrad, Saadia Ba-M’Hamed, Mohamed Nejmeddine, Mohamed Bennis

**Affiliations:** 1 Laboratory of Pharmacology, Neurobiology, Anthropobiology and Environment, Faculty of Sciences Semlalia, Cadi Ayyad University, Marrakech, Morocco; 2 Laboratory of Cell Biology and Molecular Genetics, Department of Biology, Faculty of Sciences Agadir, Ibnou Zohr University, Agadir, Morocco; 3 Higher Institute of Nursing and Health Technology Professions, Marrakech, Morocco; 4 Faculty of Medicine and Pharmacy, University Cadi Ayyad, Marrakech, Morocco; 5 School of Psychology and Neuroscience, University of St Andrews, St Andrews, United Kingdom

**Keywords:** c-Fos, dopamine D2 receptor, haloperidol, latent inhibition, mediodorsal thalamus, nucleus accumbens, prefrontal cortex

## Abstract

**Introduction:**

The mediodorsal thalamus (MD) is a higher-order thalamic nucleus that plays a central role in the maturation and function of prefrontal cortical circuits. Although developmental MD dysfunction has been implicated in cognitive deficits associated with neurodevelopmental disorders, the underlying molecular and circuit mechanisms remain poorly understood. Here, we investigated whether neonatal MD lesions produce persistent alterations in latent inhibition (LI), prefrontal dopamine D2 receptor (D2R) expression, and neuronal recruitment within the MD–prefrontal cortex (PFC)–nucleus accumbens (NAc) circuit.

**Methods:**

Bilateral excitotoxic MD lesions were induced in Sprague–Dawley rats on postnatal day 4 (MD lesion *n* = 35; MD Sham *n* = 35). In adulthood, LI was assessed using a conditioned taste aversion paradigm. Neuronal activation was evaluated by c-Fos immunohistochemistry, whereas D2R expression was quantified by immunohistochemistry, Western blotting, and quantitative PCR. The ability of the D2R antagonist haloperidol to restore LI and neuronal activity was also examined.

**Results and discussion:**

Neonatal MD lesions produced a persistent impairment of LI accompanied by reduced task-related c-Fos expression in the PFC and NAc. These functional abnormalities were associated with a selective increase in prefrontal D2R expression at the transcript, protein, and cellular levels, whereas no changes were observed in the secondary motor cortex. Importantly, haloperidol administration during stimulus pre-exposure restored LI and normalized c-Fos activation in the PFC and NAc of MD-lesion animals without affecting sham controls. These findings demonstrate that early MD dysfunction induces long-lasting remodeling of prefrontal dopaminergic signaling and disrupts recruitment of the MD–PFC–NAc circuit during selective attention. They identify altered D2R signaling as a potential mechanism linking developmental thalamic dysfunction to persistent cognitive deficits.

## Graphical abstract

**Figure:**
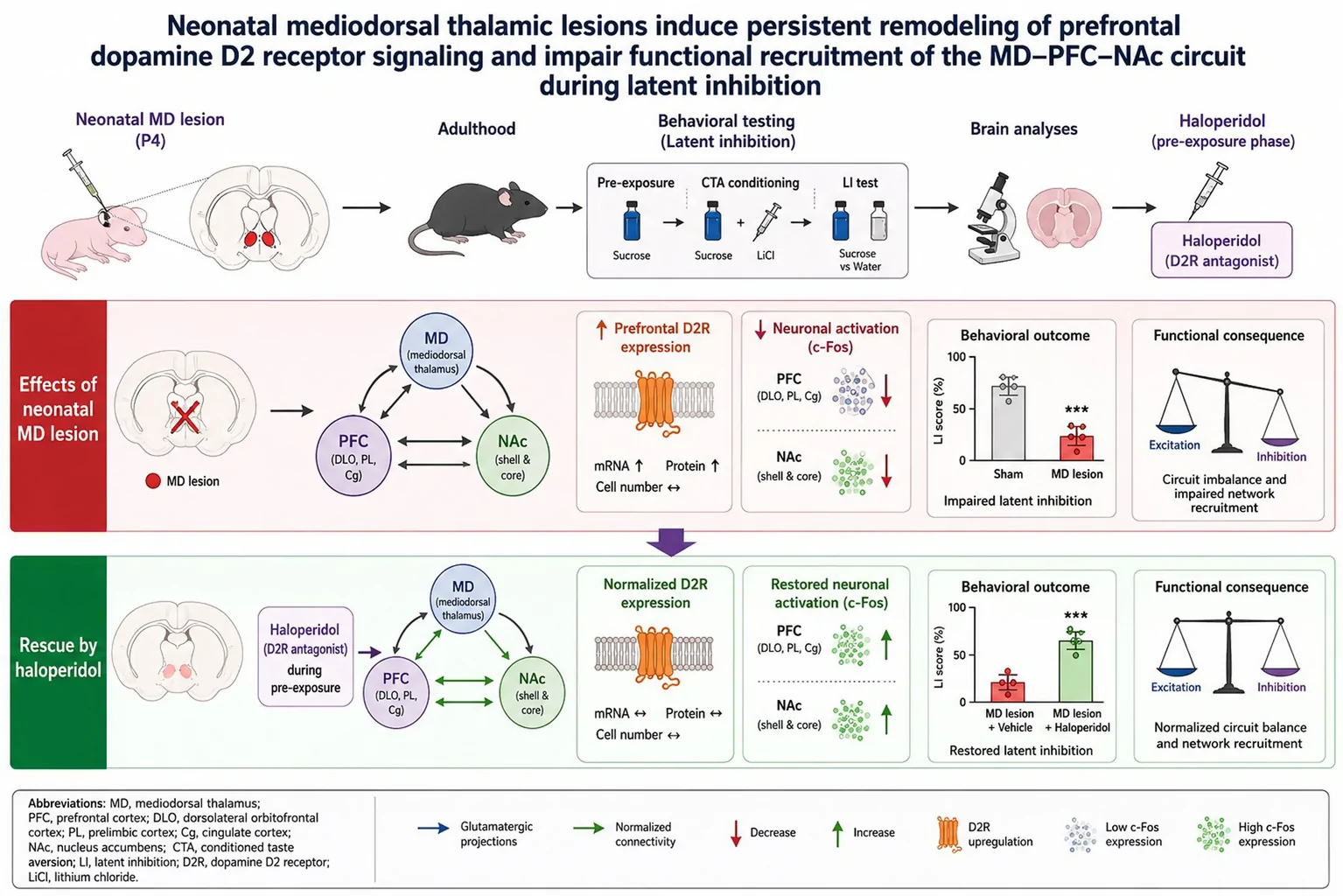

## 1 Introduction

The mediodorsal thalamus (MD) is increasingly recognized as a critical component of the neural circuitry underlying higher-order cognition. Although the thalamus has been traditionally viewed as a passive relay of sensory information (Guillery and Sherman, 2002; Jones, 2007; Sherman and Guillery, 2011), accumulating evidence indicates that higher-order thalamic nuclei actively coordinate communication between distributed cortical networks. Among these, the MD occupies a central position through its extensive reciprocal connections with the prefrontal cortex (PFC), where it contributes to executive function, working memory, behavioral flexibility, and adaptive decision-making (Mitchell et al., 2007; Parnaudeau et al., 2013, 2015; Mitchell et al., 2014; Iben-Daoudi et al., 2026).

Beyond its role in the mature brain, the MD is essential for normal cortical development. Thalamocortical projections reach the PFC around birth and continue to mature throughout postnatal life, contributing to cortical lamination, synaptic refinement, and activity-dependent plasticity (Van Eden and Uylings, 1985; Van Eden, 1986; Benes, 1989; Rios and Villalobos, 2004). During the same developmental period, corticostriatal projections linking the PFC and nucleus accumbens (NAc) also undergo extensive maturation (Chen et al., 2025), supporting coordinated development of the MD–PFC–NAc circuit. Disruption of MD activity during this critical developmental window is therefore expected to produce persistent alterations across this network rather than isolated thalamic dysfunction.

Consistent with this hypothesis, neonatal excitotoxic MD lesions produce long-lasting behavioral abnormalities, including impairments in learning, working memory, behavioral flexibility, and social interaction, together with increased stereotypic behaviors (Ouhaz et al., 2015, 2017, 2018). Similar phenotypes are observed in psychostimulant-based and neurodevelopmental models of schizophrenia (Lipska and Weinberger, 1993; Lipska et al., 2004), suggesting that developmental MD dysfunction compromises the maturation of neural circuits supporting cognition.

One mechanism through which neonatal MD lesions may impair cognitive function is persistent disruption of mesocorticolimbic dopaminergic signaling. Experimental evidence indicates that MD dysfunction reduces dopaminergic transmission within the PFC while enhancing dopamine activity in the NAc (Heidbreder et al., 2000; Fone and Porkess, 2008). Because the PFC exerts major regulatory control over mesolimbic dopamine transmission through corticostriatal pathways (Pycock et al., 1980; Deutch, 1993), altered maturation of the MD–PFC pathway may produce long-term dysregulation of dopamine signaling throughout the circuit. Dopamine D2 receptors (D2Rs) are of particular interest because they undergo marked postnatal maturation, represent the principal pharmacological targets of antipsychotic drugs, and play important roles in attentional processing and cognitive flexibility (Schneider and Koch, 2003; King et al., 2009).

Latent inhibition (LI), defined as the reduced associability of a stimulus following repeated non-reinforced exposure (Lubow and Weiner, 2010), is a well-established measure of selective attention and aberrant salience processing. Lesion and functional studies have implicated the MD, PFC, and NAc in LI expression (Gabriel et al., 1989, 1991; Weiner et al., 1996; Young et al., 2005), supporting the concept that coordinated activity within the MD–PFC–NAc circuit is required for normal attentional processing. Importantly, LI is highly sensitive to dopaminergic modulation: dopamine agonists disrupt LI, whereas D2R antagonists, including haloperidol, reliably restore or enhance LI in experimental animals and humans (Feldon and Weiner, 1991; Moser et al., 2000; Arad and Weiner, 2009; Ouhaz et al., 2014).

Despite compelling evidence implicating the MD in cognition and neurodevelopment, it remains unknown whether early MD dysfunction induces persistent remodeling of prefrontal D2R expression and alters recruitment of the MD–PFC–NAc circuit during latent inhibition. Furthermore, it is unclear whether these abnormalities remain responsive to D2R blockade in adulthood.

We therefore hypothesized that neonatal MD lesions would disrupt maturation of the MD–PFC–NAc circuit, leading to persistent remodeling of prefrontal D2R signaling, impaired neuronal recruitment during latent inhibition, and behavioral deficits that remain responsive to dopaminergic modulation. To test this hypothesis, we combined behavioral, molecular, and neuroanatomical approaches in three complementary experiments. We first assessed latent inhibition using a conditioned taste aversion paradigm while mapping task-related neuronal activation in the PFC and NAc by c-Fos immunohistochemistry. We then quantified the long-term effects of neonatal MD lesions on prefrontal D2R expression at the transcript, protein, and cellular levels. Finally, we determined whether haloperidol administered during the pre-exposure phase restored latent inhibition and normalized neuronal recruitment within the MD–PFC–NAc circuit.

## 2 Materials and methods

### 2.1 Animals

Seventy Sprague–Dawley male rats were bred at the animal facility of Cadi Ayyad University, Marrakech, Morocco. Pregnant females were housed individually under controlled temperature and humidity on a 12-h light/dark cycle with food and water available *ad libitum*. All experimental procedures complied with the European Directive 2010/63/EU and the UK Animal (Scientific Procedures) Act 1986 and were approved by the Council Committee of the Research Laboratories of the Faculty of Sciences, Cadi Ayyad University (Approval No. BA-05/2020). Every effort was made to minimize animal suffering and reduce the number of animals used.

### 2.2 Neonatal surgery

The day of birth was designated as postnatal day 0 (P0). At P4, pups (7–8 g) were anesthetized with isoflurane in 100% oxygen (4% for induction and 1.5–2% for maintenance; oxygen flow rate, 1 L/min). Buprenorphine (0.05 mg/kg, s.c.) was administered before surgery for perioperative analgesia. We then placed pups in a neonatal stereotaxic apparatus (David Kopf Instruments) under aseptic conditions and maintained them on a thermostatically controlled warming surface throughout the procedure. Animals were randomly assigned to the MD lesion or MD sham group (*n* = 35 per group).

Bilateral excitotoxic lesions of the mediodorsal thalamus were performed as previously described (Ouhaz et al., 2015). Briefly, a Hamilton microsyringe was lowered using the following stereotaxic coordinates relative to Bregma: anteroposterior (AP), −1.0 mm; mediolateral (ML), ±0.5 mm; and dorsoventral (DV), −3.5 mm. MD-lesion animals received bilateral injections of 0.5 μL N-methyl-D-aspartate [NMDA; 0.12 M in phosphate-buffered saline (PBS)] over 4 min, whereas sham animals received an equivalent volume of PBS at the same coordinates. Following surgery, the incision was sealed with surgical glue, and pups were allowed to recover on a heated pad before being returned to their dams.

Animals remained with their mothers until weaning at postnatal day 30 (P30), after which they were group-housed (five animals per cage) under standard laboratory conditions. Behavioral and molecular experiments were conducted between P53 and P70 according to the experimental timeline (Figure 1).

**Figure 1:**
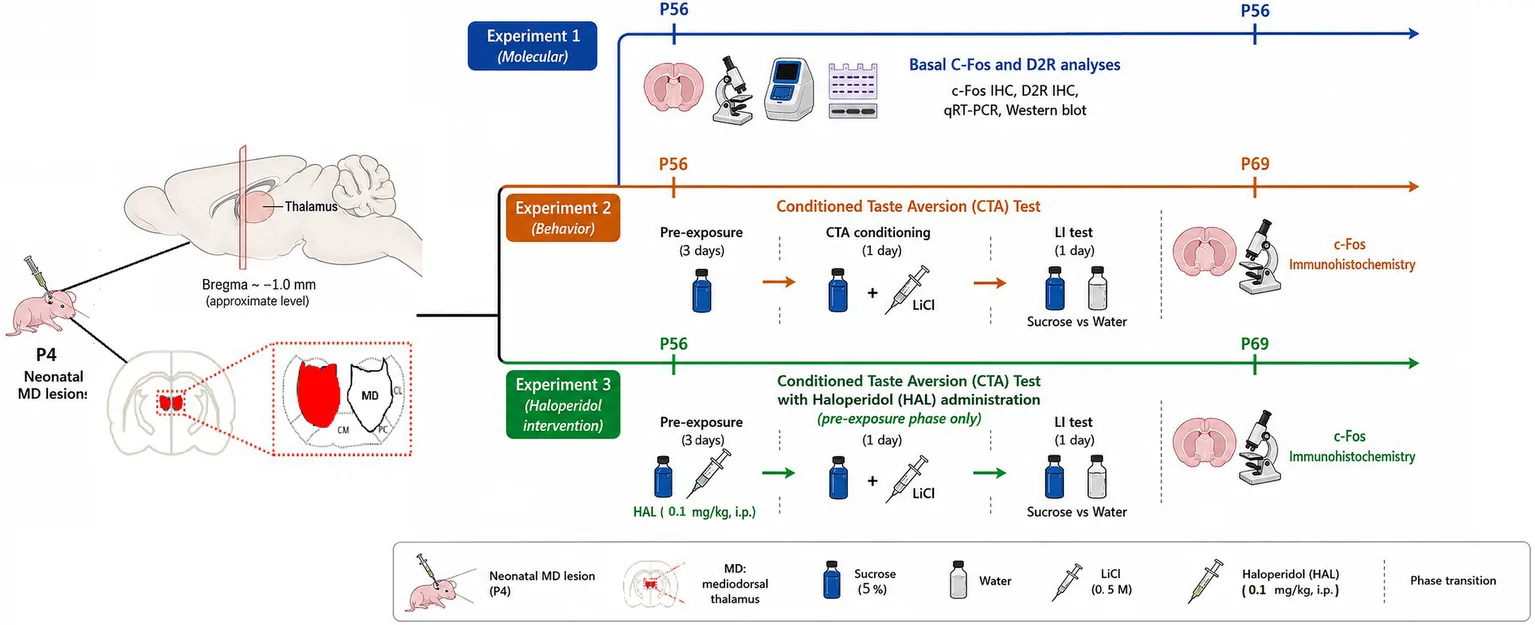
Experimental design and timeline. Neonatal mediodorsal thalamic (MD) lesions were induced on postnatal day 4 (P4). Experiment 1 assessed basal c-Fos expression and dopamine D2 receptor (D2R) expression by immunohistochemistry, quantitative real-time PCR (qRT-PCR), and Western blotting at P56. Experiment 2 evaluated latent inhibition using the conditioned taste aversion (CTA) paradigm from P56, followed by c-Fos immunohistochemistry at P69. Experiment 3 examined the effects of haloperidol administered during stimulus pre-exposure on CTA performance and task-related c-Fos expression at P69.

At the experimental endpoint, animals were deeply anesthetized by intramuscular administration of ketamine (1 mL/kg body weight; 100 mg/mL; CP-pharma) combined with xylazine (0.6 mL/kg body weight; 20 mg/mL; CP-pharma), corresponding to doses of 100 and 12 mg/kg, respectively. Adequate anesthetic depth was confirmed by the absence of the pedal-withdrawal reflex before initiation of any terminal procedure. For histological verification and immunohistochemistry, transcardial perfusion with phosphate-buffered saline (PBS), followed by 4% paraformaldehyde (PFA), was performed as a terminal procedure under deep anesthesia. Brains were subsequently collected and fixed for further processing. For quantitative PCR and Western blot analyses, deeply anesthetized animals were euthanized by decapitation; brains were rapidly removed, and the regions of interest were dissected, immediately frozen, and stored at −80 °C until RNA or protein extraction.

### 2.3 Experiment 1: effects of neonatal MD lesions on prefrontal D2 receptor expression and basal neuronal activity

The secondary motor cortex (M2), which receives minimal projections from the mediodorsal thalamus, was used as a cortical control region to assess the regional specificity of neonatal MD lesion effects.

#### 2.3.1 Quantitative real-time PCR

At postnatal day 56 (P56), a cohort of adult rats (MD sham, *n* = 5; MD lesion, *n* = 5) was deeply anesthetized with ketamine (1 mL/kg body weight; 100 mg/mL; CP-Pharma) combined with xylazine (0.6 mL/kg body weight; 20 mg/mL; CP-Pharma), corresponding to doses of 100 and 12 mg/kg, respectively, and subsequently euthanized. The prefrontal cortex (PFC) and secondary motor cortex (M2) were rapidly dissected for RNA and protein extraction.

Total RNA was isolated using the RNeasy Mini Kit (Qiagen). RNA concentration and purity were determined spectrophotometrically, and 1 μg of total RNA was reverse-transcribed using RevertAid Reverse Transcriptase (Thermo Scientific). Quantitative real-time PCR (qRT-PCR) was performed on an ABI Prism 7,300 detection system (Applied Biosystems). Gene expression was analyzed using the comparative ΔΔCt method with GAPDH as the endogenous reference gene. Reactions were performed in technical duplicates and biological triplicates. Primers were designed using Annhyb software (https://www.bioinformatics.org/annhyb/) and synthesized by Invitrogen (Thermo Fisher Scientific, UK) (Table 1).

**Table 1:** Primer sequences used for quantitative real-time PCR.

**Gene**	**Forward primer (5′–3′)**	**Reverse primer (5′–3′)**
Dopamine D2 receptor	CCC TAT GGC TTG AAG AGC CTG	AGT TGC CCT TTA GTG GAG CC
GAPDH	GGA GTC TAC TGG CGT CTT CA	ATG CCA AAG TTG TCA TGG AT

The suitability of the reference gene was evaluated in a preliminary experiment using the geNorm algorithm. Three candidate reference genes, GAPDH, TBP, and UBC, were assessed for expression stability across the experimental groups. GAPDH showed stable expression under the experimental conditions and was therefore selected as the reference gene for normalization of D2R mRNA expression.

qPCR analyses were performed using five independent animals per experimental group (*n* = 5 biological replicates/group). Each qPCR reaction was performed in technical triplicate wells, and the assay was repeated across three independent experimental runs. Technical replicate measurements were combined at the animal level, and each animal contributed a single value to the statistical analysis.

#### 2.3.2 Western blot analysis

Proteins were extracted from the same PFC and M2 tissue samples used for RNA isolation. Following RNA extraction with the RNeasy Mini Kit (Qiagen), proteins were precipitated from the lysates according to the manufacturer’s instructions, resuspended in Laemmli buffer, and quantified using the bicinchoninic acid (BCA) assay (Sigma–Aldrich).

Equal amounts of protein (30 μg) were separated on 8% SDS–polyacrylamide gels and transferred onto nitrocellulose membranes (Bio-Rad). After blocking in 5% non-fat milk in TBST, membranes were incubated overnight at 4 °C with rabbit anti-D2 receptor antibody (1:10,000; Invitrogen, PA5-109660) or mouse anti-GAPDH antibody (1:20,000; Ambion, AM4300). Membranes were then incubated with horseradish peroxidase-conjugated secondary antibodies (anti-rabbit, 1:20,000; GE Healthcare, NA934; anti-mouse, 1:40,000; GE Healthcare, NA931). Immunoreactive bands were visualized and quantified using Quantity One software (Bio-Rad), and D2R expression was normalized to GAPDH.

##### 2.3.2.1 Biological and experimental replicates

Western blot analyses were performed using samples from five independent animals per experimental group (*n* = 5 biological replicates/group). Western blot experiments were repeated across three independent experimental runs using samples derived from the same five animals per group. No within-run technical replicates were performed. Repeated experimental runs were not considered additional biological replicates, and each animal contributed a single value to the statistical analysis.

### 2.4 Experiment 2: effect of neonatal MD lesions on latent inhibition and CTA-induced neuronal activation

#### 2.4.1 Conditioned taste aversion (CTA) paradigm

A second cohort of rats (*N* = 20) was used to evaluate the effects of neonatal MD lesions on latent inhibition (LI) using a conditioned taste aversion (CTA) paradigm, as previously described by Bethus et al. (2005). Animals were housed individually in Plexiglas cages (30 × 15 × 12 cm). The conditioned stimulus consisted of a freshly prepared 5% sucrose solution, whereas the unconditioned stimulus was an intraperitoneal injection of lithium chloride (LiCl; 127 mg/kg, 0.5 M; Acros Organics).

Before behavioral testing, animals were habituated to a restricted drinking schedule by allowing access to water for 30 min daily over six consecutive days. Following habituation, rats were randomly assigned to one of four experimental groups (*n* = 5 per group): pre-exposed MD sham (PE-MD-Sham), non-pre-exposed MD sham (NPE-MD-Sham), pre-exposed MD lesion (PE-MD-lesion), and non-pre-exposed MD lesion (NPE-MD-lesion).

The pre-exposure phase consisted of three consecutive daily sessions during which pre-exposed animals received access to the 5% sucrose solution for 30 min, whereas non-pre-exposed animals received water only. Twenty-four hours after the final pre-exposure session, all animals underwent the conditioning session, during which they were allowed to consume the 5% sucrose solution for 30 min before receiving an intraperitoneal injection of LiCl to induce conditioned taste aversion.

After one recovery day, during which water was available for 30 min under the same drinking schedule, latent inhibition was evaluated using a two-bottle choice test. Animals were given simultaneous access to water and the 5% sucrose solution for 30 min, and the volumes consumed from each bottle were recorded. Greater sucrose consumption by pre-exposed animals relative to non-pre-exposed animals was considered indicative of intact latent inhibition. Following behavioral testing, brains were processed for c-Fos immunohistochemistry as described later.

### 2.5 Experiment 3: effect of haloperidol on latent inhibition and neuronal activation following neonatal MD lesions

#### 2.5.1 Haloperidol treatment and conditioned taste aversion

A third cohort of rats (*N* = 40) was used to determine whether dopamine D2 receptor blockade restores latent inhibition (LI) following neonatal MD lesions. The conditioned taste aversion (CTA) paradigm was performed exactly as described for Experiment 2, with the addition of haloperidol treatment during the pre-exposure phase.

Animals were randomly assigned to one of eight experimental groups (*n* = 5 per group) according to lesion status, pre-exposure condition, and pharmacological treatment (Table 2).

**Table 2:** Experimental groups used in Experiment 3.

Group	MD lesion	Pre-exposure	Treatment	n
NPE-MD-Sham-VEH	Sham	No	Vehicle	5
PE-MD-Sham-VEH	Sham	Yes	Vehicle	5
NPE-MD-lesion-VEH	MD lesion	No	Vehicle	5
PE-MD-lesion-VEH	MD lesion	Yes	Vehicle	5
NPE-MD-Sham-HAL	Sham	No	Haloperidol	5
PE-MD-Sham-HAL	Sham	Yes	Haloperidol	5
NPE-MD-lesion-HAL	MD lesion	No	Haloperidol	5
PE-MD-lesion-HAL	MD lesion	Yes	Haloperidol	5

Haloperidol [(0.1 mg/kg, i.p.; Sigma–Aldrich); (Ouhaz et al., 2014; Bethus et al., 2006)] or an equivalent volume of vehicle (0.9% saline) was administered 30 min before each of the three pre-exposure sessions. No injections were administered during the conditioning or testing phases. Conditioning with lithium chloride and assessment of latent inhibition using the two-bottle choice test were performed as described for Experiment 2.

Sucrose and water consumption were recorded during all phases of the CTA paradigm. Latent inhibition was evaluated by comparing sucrose consumption between pre-exposed and non-pre-exposed animals under each lesion and treatment condition. Animals were perfused 90 min after the conditioned taste aversion test, and brains were processed for c-Fos immunohistochemistry as described in Section 2.7.

### 2.6 Histological verification and image analysis

The extent and specificity of neonatal MD lesions were assessed post-mortem on cresyl violet-stained coronal sections. Animals assigned to the MD-lesion group were considered eligible for inclusion when histological examination confirmed bilateral MD lesions associated with approximately 70–80% reduction in cresyl violet-stained cell density, with minimal extension into adjacent thalamic nuclei. Animals with unilateral lesions, incomplete lesions resulting in less than 70% MD cell loss, or substantial off-target damage involving neighboring thalamic nuclei were predefined for exclusion. Sham animals were required to show an intact MD without substantial injection-related tissue damage. All animals satisfied the corresponding histological inclusion criteria; therefore, no animals were excluded on the basis of lesion location, laterality, extent, or off-target damage. Lesion verification was performed independently of subsequent behavioral, molecular, and c-Fos outcomes.

Image acquisition and quantitative analyses were performed by an investigator blinded to the experimental groups. Bright-field images were acquired using a Leica DM5000B microscope equipped with a digital camera under identical illumination, magnification, and exposure settings. Quantitative analyses were performed using ImageJ software (National Institutes of Health, Bethesda, MD, USA).

D2 receptor (D2R) immunoreactivity was quantified bilaterally in anatomically matched sections of the dorsolateral orbitofrontal (DLO), prelimbic (PL), cingulate (Cg), and secondary motor (M2) cortices according to the Paxinos and Watson rat brain atlas. For each region, the density of D2R-positive neurons (cells/mm^2^), the mean optical density of D2R immunolabeling, and the normalized D2R immunoreactivity per neuron (optical density divided by the number of D2R-positive neurons) were determined. Only neurons displaying clear somatic D2R immunoreactivity above background staining were included in the analyses.

Neuronal activation was assessed by quantifying c-Fos-positive nuclei within the DLO, PL, Cg, nucleus accumbens (NAc), and its core and shell subdivisions. Only nuclei exhibiting intense, well-defined nuclear immunoreactivity clearly distinguishable from background staining were considered positive. Cell counts were performed bilaterally on anatomically matched sections and expressed as the density of c-Fos-positive neurons (cells/mm^2^). For each animal, measurements obtained from all analyzed sections (12 sections per brain region, per animal, per marker) were averaged to generate a single value for each brain region before statistical analysis.

### 2.7 Immunohistochemistry

Free-floating coronal sections (40 μm) containing the prefrontal cortex (PFC), nucleus accumbens (NAc), and mediodorsal thalamus (MD) were collected using a freezing microtome. Every sixth section was processed for immunohistochemistry.

Sections were rinsed in 0.1 M phosphate-buffered saline (PBS; pH = 7.4), incubated in 0.3% hydrogen peroxide to quench endogenous peroxidase activity, and blocked in PBS containing 5% normal goat serum and 0.3% Triton X-100. Sections were then incubated overnight at 4 °C with either rabbit polyclonal anti-D2 receptor antibody (1:500; Invitrogen/Thermo Fisher Scientific, Cat. No. PA5-115142, reported by the manufacturer to react with rat, mouse, and human DRD2 and validated for immunohistochemical applications, including paraffin-embedded and frozen tissue) or rabbit anti-c-Fos antibody [(1:10,000; Santa Cruz Biotechnology, sc-52; RRID: AB_2106783), directed against FOS and registered for reactivity with rat, mouse, and human tissue. This antibody has been widely used for immunohistochemical detection of c-Fos].

Following primary antibody incubation, sections were incubated with biotinylated goat anti-rabbit IgG (1:500; Vector Laboratories), diluted in PBS containing 1% normal goat serum. Sections were subsequently incubated with the avidin–biotin–peroxidase complex prepared according to the manufacturer’s instructions using the Vectastain Elite ABC Kit (Vector Laboratories). Immunoreactivity was visualized using 0.05% 3,3′-diaminobenzidine (DAB). Sections were mounted, dehydrated, cleared in xylene, and coverslipped with DPX mounting medium.

To minimize staining variability, all sections from the different experimental groups were processed simultaneously under identical experimental conditions.

### 2.8 Primary outcomes and sample size determination

Primary outcomes were defined according to the principal hypothesis addressed in each experiment. In Experiment 1, the primary outcome was D2R expression in the PFC, assessed using three complementary endpoints: D2R immunoreactivity by IHC, D2R mRNA expression by qRT-PCR, and D2R protein expression by Western blot. In Experiment 2, the primary outcome was behavioral latent inhibition, assessed by sucrose consumption during the conditioned taste-aversion test and the effect of pre-exposure according to lesion status. In Experiment 3, the primary outcome was the effect of haloperidol on the pre-exposure-dependent behavioral response during the conditioned taste-aversion test. Regional c-Fos expression in Experiments 2 and 3 was considered a secondary outcome used to characterize neuronal recruitment associated with the behavioral effects.

We determined sample sizes prospectively by *a priori* power analyses using G*Power software (*GPower* 3.1.9.7) during the experimental design stage. Expected effect sizes were derived from independent previously published studies using comparable experimental paradigms and from previous studies conducted in our laboratory using neonatal lesion models (Ouhaz et al., 2014, 2015, 2017). To avoid basing sample-size estimates on the exceptionally large effects observed for some endpoints in these earlier studies, conservative effect-size assumptions were used. All calculations assumed a two-sided α level of 0.05 and statistical power of 80% (1 − *β* = 0.80).

For Experiment 1, the primary molecular comparison involved D2R expression between MD-sham and MD-lesion animals. Previous independent studies from our laboratory had demonstrated large and persistent effects of neonatal MD lesions on PFC cellular and functional measures (Ouhaz et al., 2015, 2017). A conservative standardized between-group effect of approximately Cohen’s *d* = 2.1 was therefore used for prospective planning. For a two-sided independent-samples comparison with *α* = 0.05 and 80% power, this corresponded to a minimum sample size of approximately *n* = 5 animals per group for the molecular analyses. Additional animals were included for the immunohistochemical analyses (*n* = 8/group) to increase biological replication and permit regional quantification.

For Experiment 2, which followed a 2 × 2 factorial design comprising lesion status (MD-sham vs. MD lesion) and pre-exposure (PE vs. NPE), the prospective calculation targeted the lesion × pre-exposure interaction. Based on previous latent-inhibition studies and using a conservative large interaction effect of Cohen’s *f* = 0.50, *α* = 0.05 and 80% power, the estimated minimum total sample size was *N* = 17 animals, corresponding to approximately 4–5 animals per experimental condition.

For Experiment 3, which followed a 2 × 2 × 2 factorial design comprising lesion status, pre-exposure and treatment (vehicle vs. haloperidol), the prospective calculation targeted the lesion × pre-exposure × treatment interaction. The effect-size assumption was informed by an independent previous study from our laboratory using a closely related conditioned-taste-aversion/haloperidol paradigm (Ouhaz et al., 2014), in which a large three-way interaction was observed [*F*(1,32) = 74.486, corresponding to partial η^2^ ≈ 0.699 and Cohen’s f ≈ 1.52]. To avoid basing the new experiment on this exceptionally large estimate, a substantially more conservative effect size of *f* = 0.50 was used for prospective planning. With α = 0.05 and 80% power, the resulting minimum total sample size was approximately *N* = 34 animals. The planned behavioral sample was increased beyond this minimum to allow for potential attrition and exclusions related to neonatal surgery and subsequent histological verification.

### 2.9 Randomization and allocation

Animals were allocated to experimental groups using a manual randomization procedure based on drawing lots. At P4, pups were randomly assigned within litters to either the MD-lesion or MD-sham condition by drawing identical folded allocation slips from an opaque container without replacement, while maintaining approximately balanced group sizes. At adulthood, animals were further randomized within each surgical condition to the pre-exposed (PE) or non-pre-exposed (NPE) condition using the same procedure. For Experiment 3, animals within each lesion × pre-exposure stratum were subsequently randomized to receive either vehicle (VEH) or haloperidol (HAL), again by drawing lots without replacement. Thus, treatment allocation was stratified by lesion and pre-exposure status to maintain balanced representation across the 2 × 2 × 2 factorial design. Allocation was performed before the corresponding experimental procedures and was independent of subsequent behavioral or biological outcomes (Figure 2).

**Figure 2:**
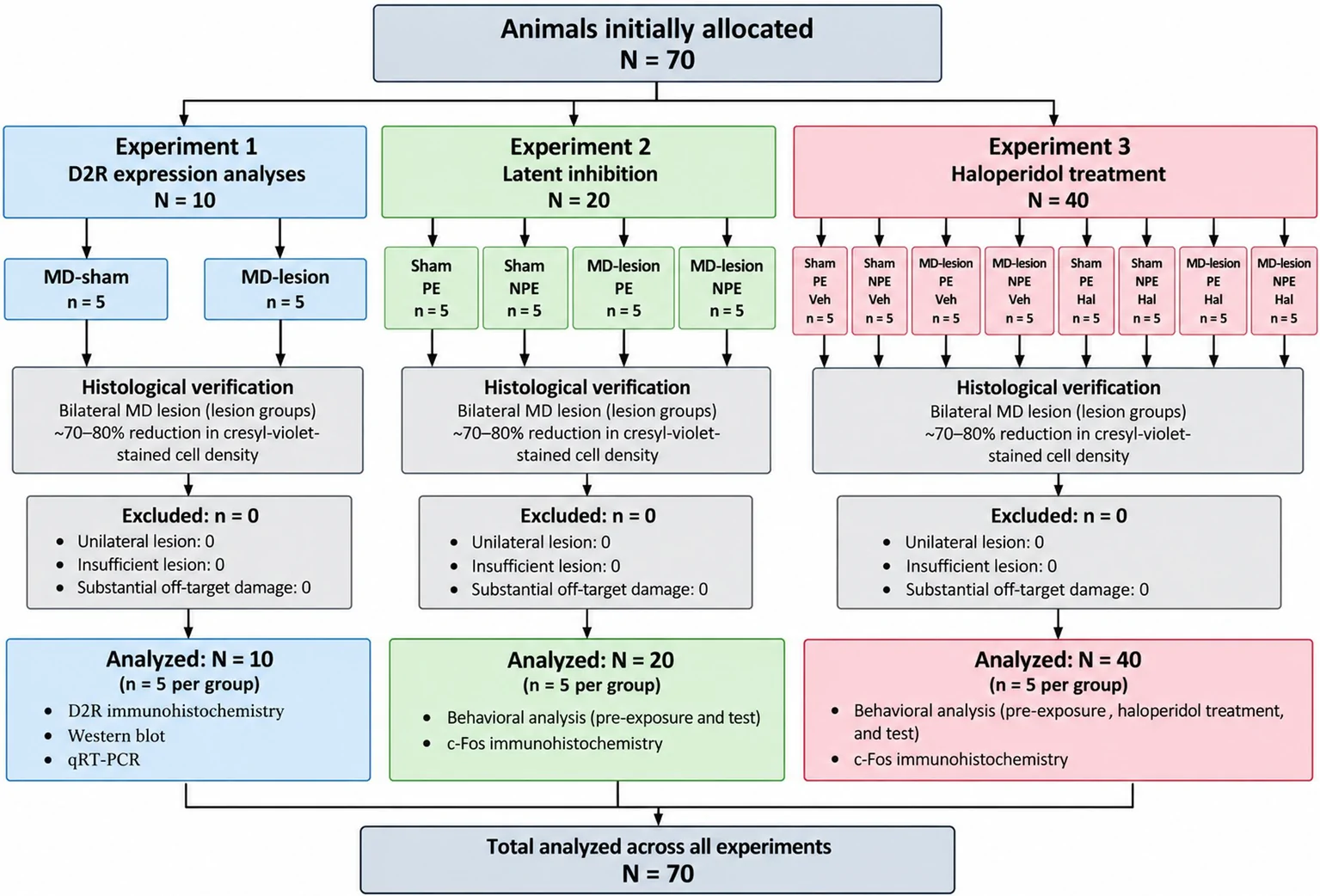
Animal allocation and experimental flow. A total of 70 rats were allocated to three independent experimental cohorts. Experiment 1 assessed D2R expression by immunohistochemistry, Western blotting, and qRT-PCR (*N* = 10; *n* = 5/group). Experiment 2 examined latent inhibition and associated c-Fos expression using a 2 × 2 design comprising lesion status (MD-sham vs. MD-lesion) and pre-exposure condition (PE vs. NPE; *N* = 20; *n* = 5/group). Experiment 3 examined the effects of haloperidol on latent inhibition and c-Fos expression using a 2 × 2 × 2 design comprising lesion status, pre-exposure condition, and treatment (vehicle vs. haloperidol; *N* = 40; *n* = 5/group). Histological verification was performed in all experimental cohorts. No animals met the predefined exclusion criteria, and all 70 animals were included in the final analyses. MD, mediodorsal thalamus; D2R, dopamine D2 receptor; PE, pre-exposed; NPE, non-pre-exposed; VEH, vehicle; HAL, haloperidol; qRT-PCR, quantitative reverse-transcription polymerase chain reaction.

### 2.10 Blinding conditions

Blinding was implemented at multiple stages of the experiments. During behavioral testing, the experimenter recording behavioral outcomes was blinded to the neonatal surgical condition (MD lesion or sham). In Experiment 3, haloperidol and vehicle solutions were prepared by an investigator not involved in drug administration or behavioral assessment and were provided in coded syringes of identical appearance. The investigator administering the injections was therefore blinded to treatment allocation, and treatment codes were not disclosed until completion of behavioral testing. Following tissue collection, samples and histological sections were assigned coded identifiers that did not reveal lesion, pre-exposure, or treatment condition. Tissue processing was performed using these coded identifiers, and image acquisition and quantitative assessment of lesion extent, D2R immunoreactivity, and c-Fos expression were performed by an investigator blinded to experimental group. Group identities were revealed only after completion of quantitative measurements. Statistical analyses were subsequently performed on the finalized dataset with knowledge of group identities.

### 2.11 Data analysis

The individual animal was considered the experimental unit for all statistical analyses. For histological and immunohistochemical outcomes, measurements obtained from multiple sections and/or microscopic fields from the same animal were aggregated to generate a single animal-level value before statistical analysis. Individual cells, sections, and microscopic fields were therefore treated as subsamples rather than independent observations. For qRT-PCR and Western blot analyses, technical measurements or replicates were similarly aggregated at the animal level, and only biological replicates (individual animals) were entered into statistical analyses. Behavioral outcomes were analyzed at the individual-animal level. Accordingly, all reported *n* values represent the number of independent animals included in each analysis.

Statistical analyses were performed using SigmaPlot 11.0 (Systat Software Inc., San Jose, CA, USA). Data are expressed as mean ± SEM. Prior to parametric testing, statistical assumptions were evaluated using SigmaPlot’s automatic normality and equal-variance procedures. Normality was assessed using the Shapiro–Wilk test, and homogeneity of variance was assessed using the Brown–Forsythe test. Given the limited sensitivity of formal normality tests at small sample sizes, particularly for molecular analyses with *n* = 5 animals per group, Shapiro–Wilk results were interpreted cautiously and were not considered in isolation as evidence of normality. When the assumptions for parametric analysis were satisfied, parametric tests were applied; when these assumptions were not satisfied, the corresponding non-parametric test was used.

Comparisons between two independent groups were performed using an unpaired Student’s *t*-test or the Mann–Whitney *U* test, as appropriate. The effects of lesion, pre-exposure, and haloperidol treatment were evaluated using two-way, three-way, or repeated-measures ANOVA, according to the experimental design. For outcomes measured repeatedly in the same animals across sessions, animal identity was specified as the subject factor, with session/time entered as the within-subject factor and the relevant experimental conditions entered as between-subject factors. Thus, repeated observations from the same animal were modeled as within-animal measurements rather than independent observations. When significant main effects or interactions were identified, Holm–Šídák *post hoc* tests were used for multiple pairwise comparisons. For repeated-measures analyses, sphericity was assessed using Mauchly’s test, and Greenhouse–Geisser corrections were applied when the sphericity assumption was violated.

In addition to *p*-values, effect sizes and 95% confidence intervals (CIs) were reported for the principal behavioral and molecular outcomes. For independent two-group comparisons, effect sizes were expressed as Hedges’ *g*, incorporating the small-sample correction, with corresponding 95% CIs. For factorial ANOVA analyses, effect sizes were expressed as partial eta-squared (ηp^2^) for the relevant main effects and interactions. For the principal behavioral comparisons, mean differences with 95% CIs were additionally reported where appropriate. All effect-size calculations were based on animal-level observations.

All statistical tests were two-tailed, and differences were considered statistically significant at *p* < 0.05.

## 3 Results

### 3.1 Lesion verification

Neonatal NMDA lesions produced a marked and selective reduction in neuronal density within the mediodorsal thalamic nucleus (MD; Figures 3A–C). Quantitative analysis confirmed substantially lower MD neuronal density in MD lesion animals than in MD sham controls [t(8) = 20.41, *p* < 0.001, Cohen’s d = 12.91, 95% CI (7.09, 18.67)]. Histological examination verified accurate lesion placement. Consistent with the anatomical selectivity of the lesions, neuronal density in adjacent thalamic nuclei, including the centrolateral (CL), centromedial (CM), paraventricular thalamic (PV), and habenular (LHeb) nuclei, did not differ significantly between MD-lesion and MD sham animals (all *p* > 0.05; Figures 3A–C).

**Figure 3:**
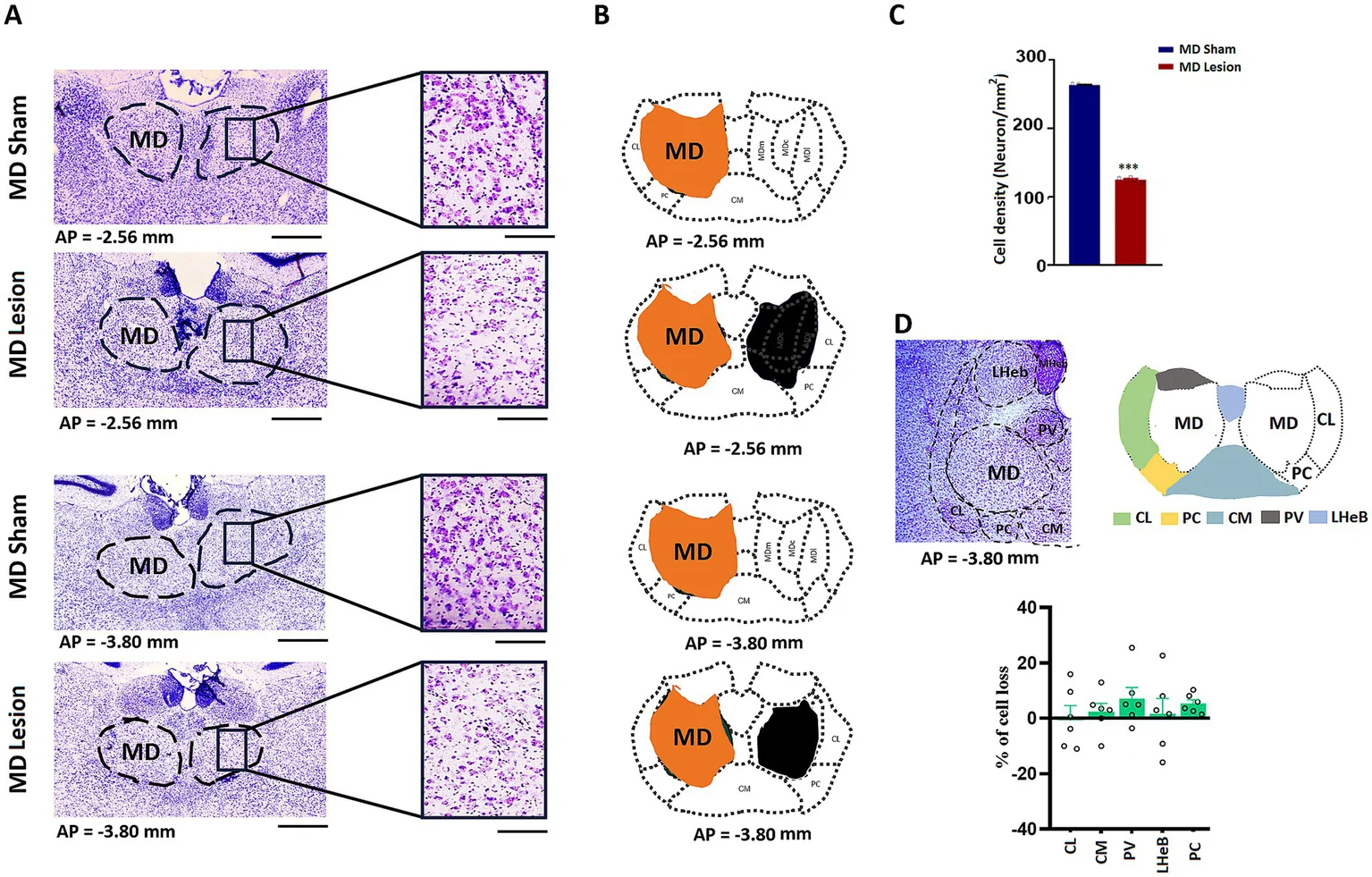
Neonatal NMDA lesions produced selective loss of cresyl-violet stained cells within the mediodorsal thalamus. **(A)** Representative photomicrographs showing lesion placement and reduced cresyl violet-stained cell density within the MD. Scale bars: 15 and 300 μm, as indicated. **(B)** Schematic reconstructions illustrating lesion extent across representative coronal levels. **(C)** Cresyl violet-stained cell density in MD-sham and MD-lesion rats. **(D)** Neuronal loss within the targeted MD and neuronal density in adjacent thalamic nuclei. Data are presented as mean ± SEM. ***p < 0.001 versus MD sham. CL, centrolateral nucleus; CM, centromedial nucleus; PC, paracentral nucleus; PV, paraventricular thalamic nucleus; LHeb, lateral habenula; MHeb, medial habenula.

### 3.2 Experiment 1. Neonatal MD lesions induce selective prefrontal D2R remodeling and reduce basal neuronal activity

#### 3.2.1 Neonatal MD lesions selectively increased prefrontal D2R expression

Early MD lesions did not significantly alter the number of D2R-immunopositive neurons in any of the examined prefrontal cortex (PFC) subregions, including the dorsolateral orbitofrontal cortex [DLO; t(8) = −0.734, *p* = 0.475, Hedges’ g = −0.42, 95% CI (−1.54, 0.73)], prelimbic cortex [PL; t(8) = 1.994, *p* = 0.066, Hedges’ g = 0.94, 95% CI (−0.16, 2.01)], or cingulate cortex [Cg; t(8) = −0.155, *p* = 0.879, Hedges’ g = 0.07, 95% CI (−0.98, 1.11); Figure 4B]. Thus, neonatal MD lesions were not associated with a statistically significant change in the number of D2R-immunopositive neurons within the examined PFC regions.

**Figure 4:**
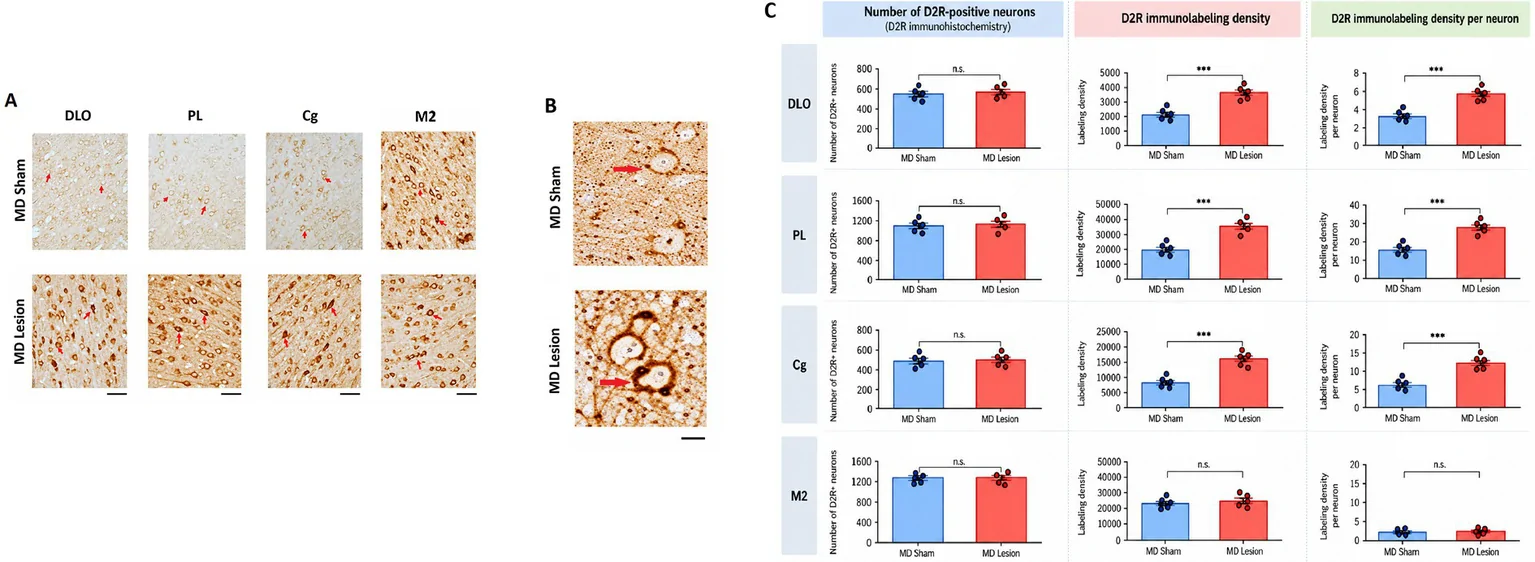
Neonatal MD lesions selectively increased D2R immunoreactivity in the prefrontal cortex. **(A)** Representative photomicrographs of D2R immunolabeling in the dorsolateral orbitofrontal (DLO), prelimbic (PL), cingulate (Cg), and secondary motor (M2) cortices of MD-sham and MD-lesion rats; arrows indicate D2R-immunopositive neurons. Scale bar: 200 μm. **(B)** High-magnification representative photomicrographs of D2R immunolabeling in the prelimbic (PL) of MD-sham and MD-lesion rats. Scale bar: 10 μm. **(C)** Density of D2R-positive neurons. **(C)** D2R labeling density. **(D)** D2R labeling density normalized to the number of D2R-positive neurons. Data are presented as mean ± SEM (*n* = 5 per group). Group comparisons were performed using two-tailed independent-samples *t* tests or Mann–Whitney *U* tests, as appropriate. *n.s* > 0.05; ***p < 0.001 versus MD sham.

In contrast, D2R immunolabeling density was markedly increased following neonatal MD lesions. Compared with sham animals, MD-lesion rats exhibited significantly greater D2R labeling density in the DLO [t(8) = −12.982, *p* < 0.001, Hedges’ g = −7.42, 95% CI (−11.13, −3.67)], PL [t(8) = −12.982, *p* < 0.001, Hedges’ g = −7.42, 95% CI (−11.13, −3.67)], and Cg [t(8) = −9.549, *p* < 0.001, Hedges’ g = −5.45, 95% CI (−8.28, −2.59); Figure 4C].

Consistent with these findings, D2R labeling density normalized to the number of D2R-immunopositive neurons was also significantly increased in MD-lesion animals in the DLO [t(8) = −10.567, *p* < 0.001, Hedges’ g = −6.04, 95% CI (−9.11, −2.94)], PL [t(8) = −12.601, *p* < 0.001, Hedges’ g = −7.20, 95% CI (−10.81, −3.55)], and Cg [t(8) = −8.204, *p* < 0.001, Hedges’ g = −4.69, 95% CI (−7.19, −2.16); Figure 4D]. Together, these findings indicate that neonatal MD lesions were associated with increased D2R immunoreactivity within D2R-positive PFC neurons rather than an increase in the number of D2R-immunopositive neurons.

These immunohistochemical findings were corroborated by biochemical analyses. Western blot analysis revealed markedly increased D2R protein expression in the PFC of MD-lesion animals compared with MD-sham controls [D2R/GAPDH ratio; *t*(8) = 6.604, *p* < 0.001, Hedges’ *g* = 3.77, 95% CI (0.686, 1.423); Figure 4C]. In contrast, D2R protein expression in M2 did not differ between MD-lesion and MD-sham animals [*t*(8) = −0.066, *p* = 0.949, Hedges’ *g* = −0.04, 95% CI (−0.051, 0.048); Figure 5C].

**Figure 5:**
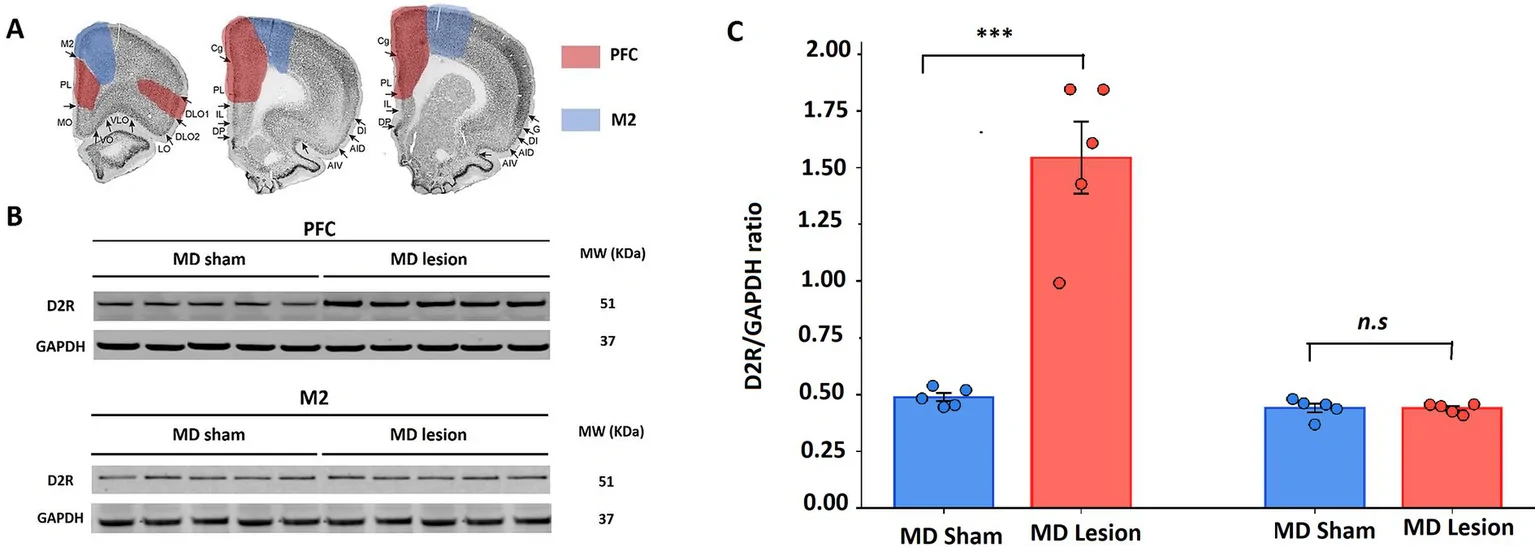
Neonatal MD lesions increased D2R protein expression in the prefrontal cortex. **(A)** Coronal sections illustrating the prefrontal cortex (PFC) and secondary motor cortex (M2) regions sampled for Western blot analysis. **(B)** Representative D2R and GAPDH immunoblots from the PFC and M2 of MD-sham and MD-lesion rats. **(C)** Densitometric quantification of D2R normalized to GAPDH. Data are presented as mean ± SEM (*n* = 5 per group) and were analyzed using two-tailed independent-samples *t*-tests. *n.s* > 0.05; ***p < 0.001 versus MD sham.

Quantitative RT-PCR similarly showed increased D2R mRNA expression in the PFC of MD-lesion animals [*t*(8) = 7.147, *p* < 0.001, Hedges’ *g* = 4.08, 95% CI (1.80, 6.31)], whereas no significant difference was detected in M2 (Mann–Whitney *U* = 8.000, *p* = 0.421, rank-biserial correlation |*r*rb| = 0.36; Figures 6A,B).

**Figure 6:**
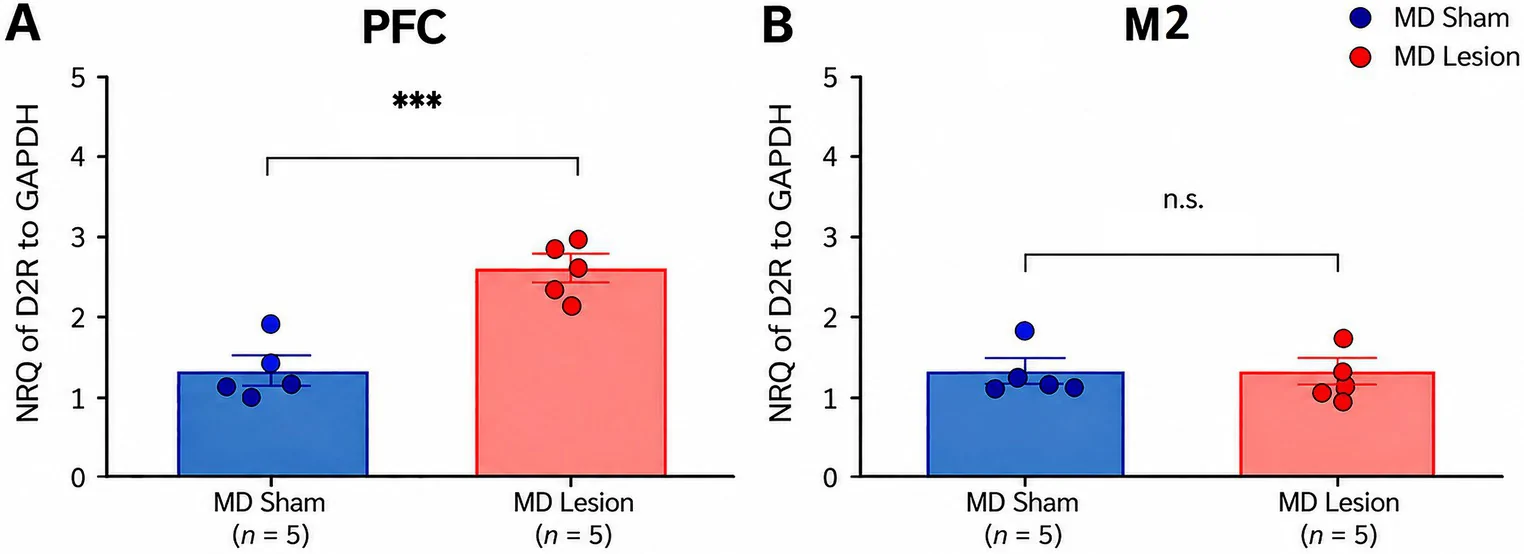
Neonatal MD lesions increased D2R mRNA expression in the prefrontal cortex. Relative D2R mRNA expression normalized to GAPDH in the **(A)** PFC and **(B)** M2 of MD-sham and MD-lesion rats. Data are presented as mean ± SEM (*n* = 5 per group). Group differences were assessed using a two-tailed independent-samples *t*-test for the PFC and a Mann–Whitney *U* test for M2. *n.s* > 0.05; ***p < 0.001 versus MD sham.

Consistent with the biochemical findings, immunohistochemical analyses detected no significant lesion-associated differences in the number of D2R-positive neurons, D2R labeling density, or normalized D2R labeling density in M2 (all *p* > 0.05; Figures 4B–D). Together, these convergent findings indicate that the persistent increase in D2R expression following neonatal MD lesions was detected in the PFC but not in the M2 control region.

#### 3.2.2 Neonatal MD lesions selectively reduced basal prefrontal c-Fos expression

Basal c-Fos expression was quantified in the dorsolateral orbitofrontal cortex (DLO), prelimbic cortex (PL), anterior cingulate cortex (Cg), and secondary motor cortex (M2). Neonatal MD lesions were associated with a marked reduction in basal c-Fos expression across all three prefrontal regions. Compared with sham animals, MD-lesion rats exhibited significantly lower c-Fos expression in the DLO [*t*(8) = 90.394, *p* < 0.001, Hedges’ *g* = 51.64, 95% CI (26.92, 76.47)], PL [*t*(8) = 74.152, *p* < 0.001, Hedges’ *g* = 42.36, 95% CI (22.08, 62.74)], and Cg [*t*(8) = 64.788, *p* < 0.001, Hedges’ *g* = 37.01, 95% CI (19.28, 54.82); Figures 7A–D]. In contrast, basal c-Fos expression in the M2 control region did not differ significantly between MD-lesion and sham animals [*t*(8) = −0.615, *p* = 0.555, Hedges’ *g* = −0.35, 95% CI (−1.47, 0.79); Figures 7A–D]. Thus, neonatal MD lesions were associated with a pronounced and regionally selective reduction in basal c-Fos expression within the DLO, PL, and Cg, while basal expression in M2 remained preserved.

**Figure 7:**
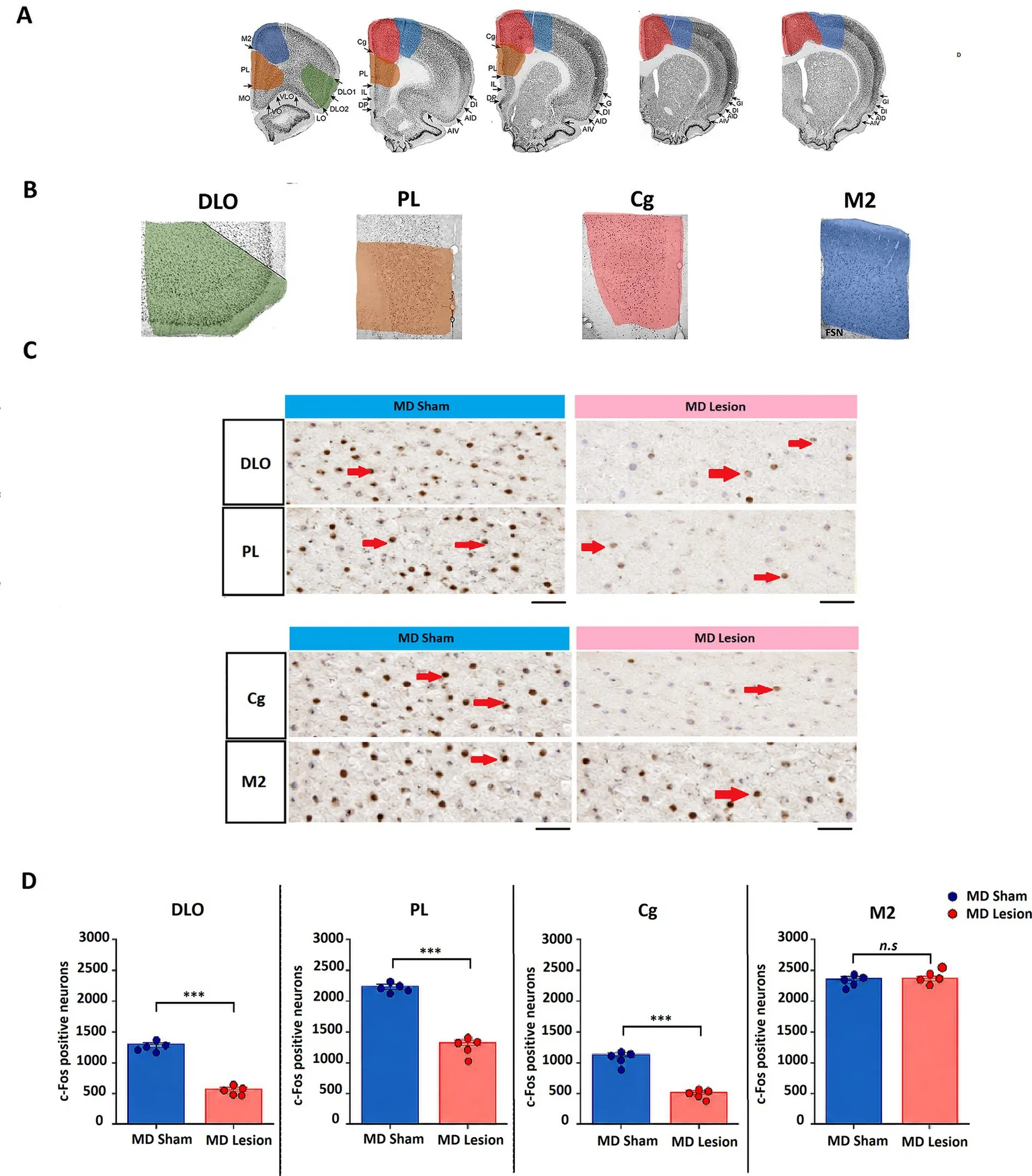
Neonatal MD lesions reduced basal c-Fos expression selectively within prefrontal cortical regions. **(A)** Coronal sections illustrating the cortical regions of interest. **(B)** Representative delineation of the DLO, PL, Cg, and M2 regions used for quantification. **(C)** Representative photomicrographs of basal c-Fos immunolabeling in MD-sham and MD-lesion rats; arrows indicate c-Fos-positive nuclei. **(D)** Density of c-Fos-positive nuclei in the DLO, Cg, PL, and M2. Scale bar: 300 μm. Data are presented as mean ± SEM (*n* = 5 per group) and were analyzed using two-tailed independent-samples *t* tests. *n.s* > 0.05; ***p < 0.001 versus MD sham.

### 3.3 Experiment 2. Neonatal MD lesions impair latent inhibition in the conditioned taste aversion paradigm

#### 3.3.1 Total liquid consumption during the conditioned taste aversion paradigm

Total liquid consumption was monitored throughout the conditioned taste aversion (CTA) paradigm to determine whether neonatal MD lesions produced nonspecific alterations in fluid intake (Figure 8A). At baseline, liquid consumption was comparable across groups, with no significant main effect of lesion status [*F*(1, 16) = 0.093, *p* = 0.763, ηp^2^ = 0.003, 95% CI (0.000, 0.13)] or pre-exposure condition [*F*(1, 16) = 0.779, *p* = 0.384, ηp^2^ = 0.024, 95% CI (0.000, 0.20)].

**Figure 8:**
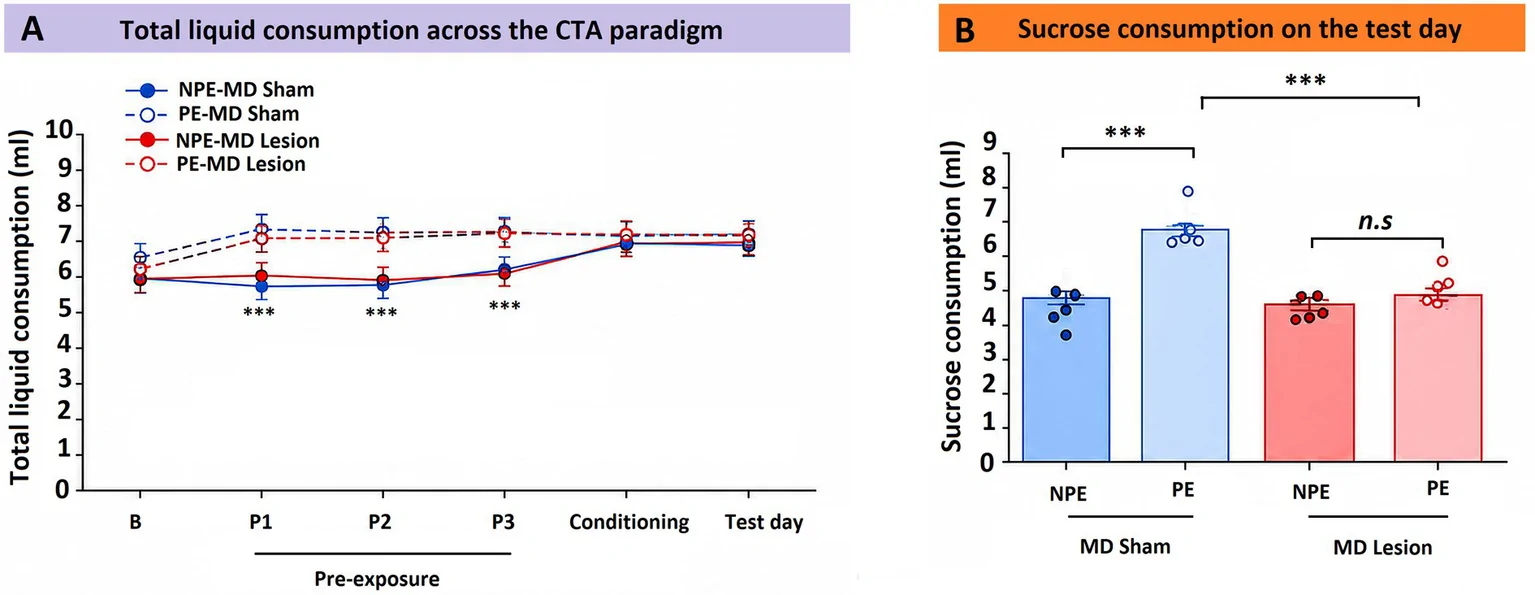
Neonatal MD lesions disrupted latent inhibition in the conditioned taste aversion paradigm. **(A)** Total liquid consumption across baseline **(B)**, pre-exposure sessions (P1-P3), conditioning (Cond), and testing (Test) in non-pre-exposed (NPE) and pre-exposed (PE) MD-sham and MD-lesion rats. **(B)** Sucrose consumption during the test session. Data are presented as mean ± SEM (*n* = 5 per group). *n.s* > 0.05; ***p < 0.001.

During the three pre-exposure sessions, a repeated-measures ANOVA revealed a significant effect of pre-exposure [*F*(1, 16) = 267.875, *p* < 0.001, ηp^2^ = 0.944, 95% CI (0.871, 0.969)], whereas MD lesion had no significant effect [*F*(1, 16) = 0.027, *p* = 0.871, ηp^2^ = 0.002, 95% CI (0.000, 0.171)]. *Post hoc* analyses confirmed that PE animals consumed more liquid than NPE animals throughout the pre-exposure phase, irrespective of lesion status (all *p* < 0.001), whereas no significant differences were detected between sham and MD-lesion animals within either pre-exposure condition (all *p* > 0.05; Figure 8A).

During conditioning, there was no significant main effect of lesion status [*F*(1, 16) = 3.227, *p* = 0.082, ηp^2^ = 0.092, 95% CI (0.000, 0.310)] or pre-exposure condition [*F*(1, 16) = 1.882, *p* = 0.180, ηp^2^ = 0.056, 95% CI (0.000, 0.260)]. Similarly, total liquid consumption on the test day was not significantly affected by lesion status [*F*(1, 16) = 2.268, *p* = 0.142, ηp^2^ = 0.066, 95% CI (0.000, 0.276)] or pre-exposure condition [*F*(1, 16) = 0.114, *p* = 0.738, ηp^2^ = 0.004, 95% CI (0.000, 0.132); Figure 8A].

Within-group repeated-measures analyses further showed similar temporal patterns in sham and MD-lesion animals. NPE animals maintained baseline levels of liquid consumption during the pre-exposure phase and exhibited increased intake during the conditioning and test sessions (all *p* < 0.001). In contrast, PE animals showed increased consumption from the first pre-exposure session onward, which remained elevated during conditioning and testing (all *p* < 0.001). Together, these findings indicate that neonatal MD lesions did not produce a generalized alteration in fluid consumption during the CTA paradigm, supporting the interpretation that the lesion-related differences observed in LI were not attributable to nonspecific changes in overall drinking behavior.

#### 3.3.2 Early MD lesions disrupt latent inhibition of conditioned taste aversion

Sucrose consumption on the test day was analyzed using a two-way ANOVA with lesion status and pre-exposure as between-subject factors. The analysis revealed significant main effects of lesion [*F*(1, 16) = 59.244, *p* < 0.001, ηp^2^ = 0.787, 95% CI (0.523, 0.874)] and pre-exposure [*F*(1, 16) = 38.384, *p* < 0.001, ηp^2^ = 0.706, 95% CI (0.389, 0.829)], together with a significant lesion × pre-exposure interaction [*F*(1, 16) = 28.133, *p* < 0.001, ηp^2^ = 0.637, 95% CI (0.287, 0.793)], indicating that the effect of pre-exposure on sucrose consumption depended on lesion status (Figure 8B).

In sham animals, PE rats consumed significantly more sucrose than NPE rats [*p* < 0.001, hedges’ g = 3.64, 95% CI (1.53, 5.69)], consistent with robust latent inhibition. In contrast, sucrose consumption did not differ significantly between PE and NPE MD-lesion animals [*p* = 0.534, hedges’ g = 0.29, 95% CI (−0.85, 1.41)], indicating disruption of the normal pre-exposure-dependent increase in sucrose consumption. MD-lesion animals also consumed significantly less sucrose than sham animals under the PE condition [*p* < 0.001, hedges’ g = 4.04, 95% CI (1.77, 6.25)], whereas the difference under the NPE condition was smaller [*p* = 0.032, hedges’ g = 0.99, 95% CI (−0.25, 2.18); Figure 8B].

Together, these findings indicate that neonatal MD lesions disrupted latent inhibition, as evidenced by the loss of the normal pre-exposure-dependent increase in sucrose consumption. In conjunction with the absence of a generalized lesion-related reduction in total fluid intake, this pattern argues against nonspecific hypodipsia as an explanation for the behavioral effect.

#### 3.3.3 Early MD lesions alter CTA-induced neuronal recruitment in the prefrontal cortex

##### 3.3.3.1 Dorsolateral orbitofrontal cortex c-Fos

In the DLO, two-way ANOVA revealed significant main effects of MD lesion [*F*(1,16) = 144.231, *p* < 0.001, ηp^2^ = 0.900, 95% CI (0.780, 0.944)] and pre-exposure [*F*(1,16) = 117.245, *p* < 0.001, ηp^2^ = 0.880, 95% CI (0.738, 0.933)], together with a significant lesion × pre-exposure interaction [*F*(1,16) = 150.979, *p* < 0.001, ηp^2^ = 0.904, 95% CI (0.788, 0.947); Figure 9C]. In sham animals, c-Fos density did not differ significantly between PE and NPE conditions (*p* > 0.05). In contrast, PE-MD-lesion rats showed markedly lower c-Fos density than both NPE-MD-lesionrats and PE-MD-sham controls (both *p* < 0.001), whereas NPE-MD-lesion and NPE-MD-sham animals did not differ significantly (*p* > 0.05). Thus, the effect of neonatal MD lesions on DLO recruitment was strongly dependent on pre-exposure condition, with reduced DLO c-Fos expression specifically observed in PE MD-lesion animals.

**Figure 9:**
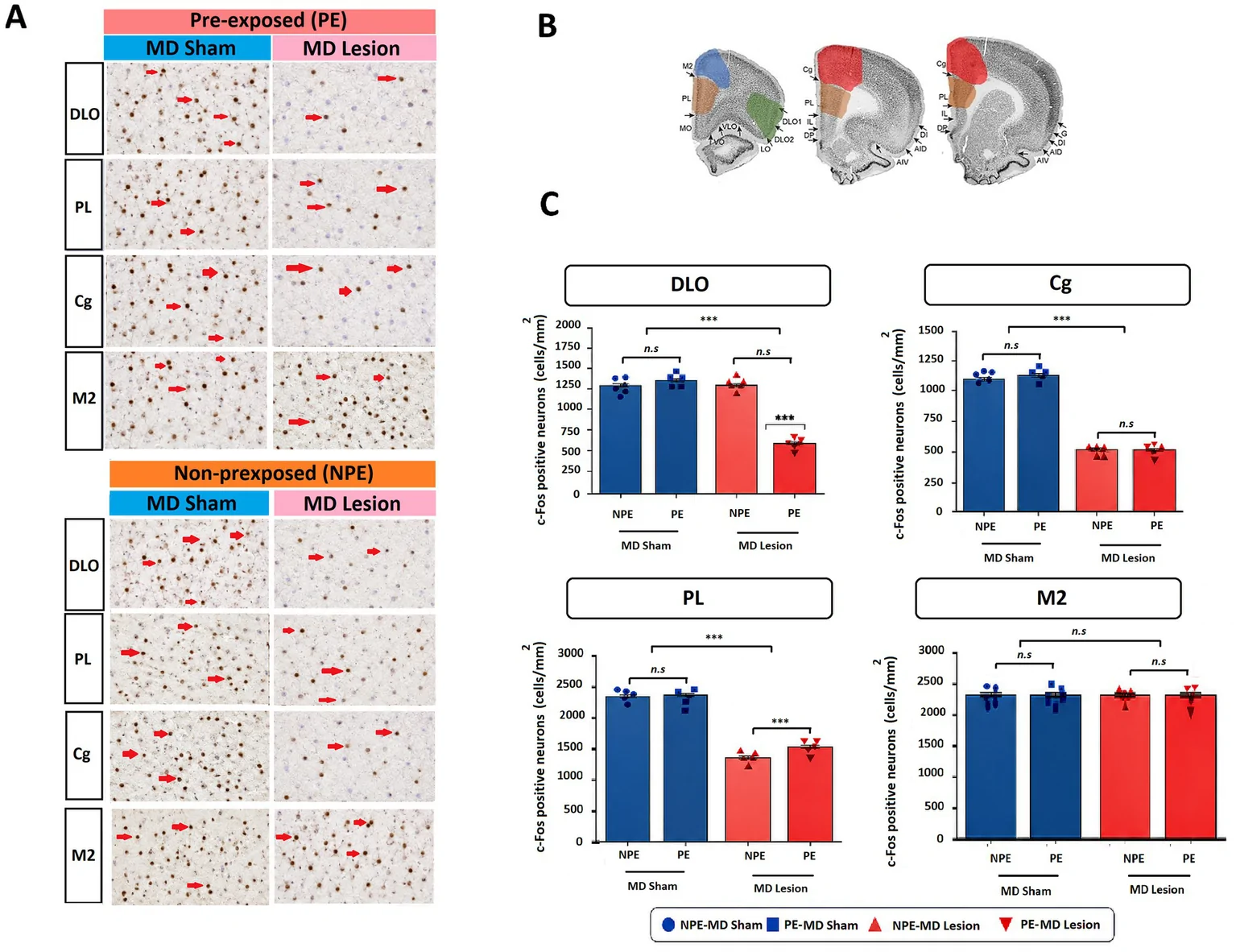
Neonatal MD lesions altered CTA-induced neuronal recruitment within the prefrontal cortex. **(A)** Representative photomicrographs of c-Fos immunolabeling in the DLO, PL, Cg, and M2 after CTA testing; arrows indicate c-Fos-positive nuclei. **(B)** Low-magnification coronal sections showing the regions used for quantification. **(C)** Density of c-Fos-positive nuclei in each cortical region. Scale bar: 40 μm. Data are presented as mean ± SEM (*n* = 5 per group) and were analyzed using two-way ANOVA (lesion × pre-exposure), followed by Holm-Šídák *post hoc* comparisons; *n.s* > 0.05; ***p < 0.001.

##### 3.3.3.2 Prelimbic cortex c-Fos

In the PL, two-way ANOVA revealed significant effects of pre-exposure [*F*(1,16) = 62.989, *p* < 0.001, ηp^2^ = 0.797, 95% CI (0.563, 0.888)] and MD lesion [*F*(1,16) = 8,464.167, *p* < 0.001, ηp^2^ = 0.998, 95% CI (0.996, 0.999)], as well as a significant pre-exposure × lesion interaction [*F*(1,16) = 16.852, *p* < 0.001, ηp^2^ = 0.513, 95% CI (0.145, 0.724)]. Holm–Šídák post-hoc comparisons showed similar c-Fos density in PE-MD-sham compared with NPE-MD-sham rats (*t* = 1.709, *p* = 0.051). However, PE-MD-lesion rats exhibited higher c-Fos density than NPE-MD-lesion rats (*t* = 8.515, *p* < 0.001), while c-Fos density was markedly lower in PE-MD-lesion than PE-MD-sham rats (*t* = 69.430, *p* < 0.001; Figure 9C).

##### 3.3.3.3 Cingulate cortex c-Fos

C-Fos expression in the cg was predominantly determined by lesion status and was not modulated by pre-exposure. Two-way ANOVA revealed a strong main effect of MD lesion [*F*(1,16) = 166.257, *p* < 0.001, ηp^2^ = 0.912, 95% CI (0.801, 0.951)], whereas neither the main effect of pre-exposure [*F*(1,16) = 1.966, *p* = 0.180, ηp^2^ = 0.109, 95% CI (0.000, 0.384)] nor the lesion × pre-exposure interaction [*F*(1,16) = 0.214, *p* = 0.650, ηp^2^ = 0.013, 95% CI (0.000, 0.262)] was significant (Figure 9C). Consistent with this pattern, c-Fos density was markedly lower in MD-lesion than sham animals under both PE and NPE conditions (both *p* < 0.001), whereas PE and NPE conditions did not differ significantly within either lesion group.

##### 3.3.3.4 Secondary motor cortex c-Fos

In contrast, neonatal MD lesions did not significantly alter c-Fos expression in the secondary motor cortex (M2). Two-way ANOVA revealed no significant main effect of lesion [*F*(1,16) = 0.592, *p* = 0.453, ηp^2^ = 0.036, 95% CI (0.000, 0.306)] or pre-exposure [*F*(1,16) = 2.189, *p* = 0.158, ηp^2^ = 0.120, 95% CI (0.000, 0.398)]. Thus, unlike the prefrontal regions examined, M2 showed no statistically detectable lesion- or pre-exposure-related changes in c-Fos expression, consistent with regional specificity of the observed prefrontal alterations (Figure 9C).

#### 3.3.4 Early MD lesions alter CTA-induced neuronal recruitment in the nucleus accumbens

##### 3.3.4.1 Basal neuronal activity in the nucleus accumbens is preserved following neonatal MD lesions

Basal c-Fos expression was assessed in the whole nucleus accumbens (NAc) and its shell and core subdivisions. Under basal conditions, neonatal MD lesions did not significantly alter c-Fos expression in the NAc shell (Mann–Whitney *U* = 10.500, *p* = 0.690, |*r*rb| = 0.16), NAc core (Mann–Whitney *U* = 10.500, *p* = 0.690, |*r*rb| = 0.16), or whole NAc [*t*(8) = 0.211, *p* = 0.838, Hedges’ *g* = 0.12, 95% CI (−0.99, 1.22); Figures 10A–C,E]. These findings indicate that neonatal MD lesions were not associated with a statistically detectable alteration in basal accumbal c-Fos expression.

**Figure 10:**
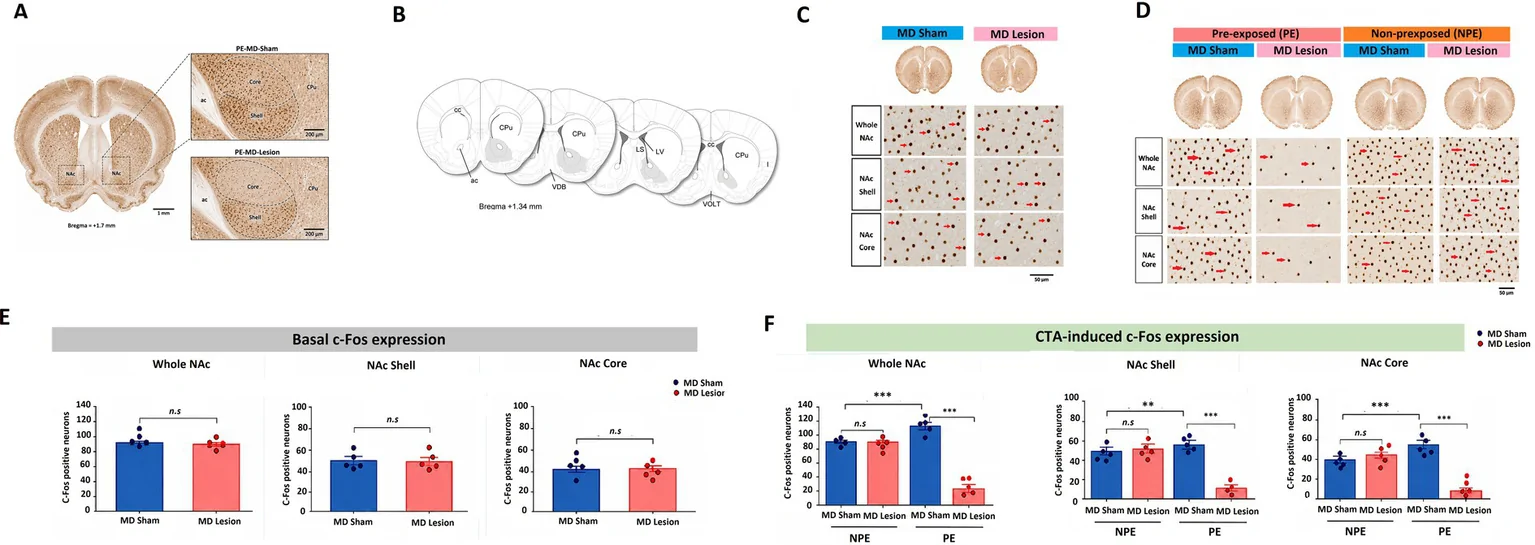
Neonatal MD lesions preserved basal accumbal c-Fos expression but disrupted CTA-induced neuronal recruitment. **(A)** Coronal sections showing the nucleus accumbens (NAc) shell and core regions used for quantification. **(B)** Schematic reconstructions illustrating brain sections involved in the quantification of accumbal c-Fos expression relative to Bregma. **(C)** Representative photomicrographs of basal c-Fos immunolabeling in the NAc shell and core of MD-sham and MD-lesion rats; arrows indicate c-Fos-positive nuclei. **(D)** Representative photomicrographs of CTA-induced c-Fos immunolabeling in NPE and PE MD-sham and MD-lesion rats. **(E)** Basal c-Fos-positive nuclear density in the whole NAc, shell, and core. **(F)** CTA-induced c-Fos-positive nuclear density in the whole NAc, shell, and core. Scale bars: 40 μm. Data are presented as mean ± SEM (*n* = 5 per group). Basal values were analyzed using two-tailed independent-samples *t* tests or Mann–Whitney U tests, as appropriate. CTA-induced values were analyzed using two-way ANOVA (lesion × pre-exposure), followed by Holm-Šídák *post hoc* comparisons. *n.s* > 0.05; ***p < 0.001.

##### 3.3.4.2 Neonatal MD lesions alter pre-exposure-dependent neuronal recruitment in the nucleus accumbens

We next examined CTA-associated neuronal recruitment in the whole NAc and its shell and core subdivisions. In the whole NAc, two-way ANOVA revealed significant main effects of lesion [*F*(1,16) = 273.001, *p* < 0.001, ηp^2^ = 0.945, 95% CI (0.875, 0.969)] and pre-exposure [*F*(1,16) = 71.290, *p* < 0.001, ηp^2^ = 0.817, 95% CI (0.599, 0.899)], together with a significant lesion × pre-exposure interaction [*F*(1,16) = 270.601, *p* < 0.001, ηp^2^ = 0.944, 95% CI (0.874, 0.969)].

Similar effects were observed in the NAc shell, with significant main effects of lesion [*F*(1,16) = 82.008, *p* < 0.001, ηp^2^ = 0.837, 95% CI (0.640, 0.910)] and pre-exposure [*F*(1,16) = 26.721, *p* < 0.001, ηp^2^ = 0.626, 95% CI (0.272, 0.787)], together with a significant lesion × pre-exposure interaction [*F*(1,16) = 73.778, *p* < 0.001, ηp^2^ = 0.822, 95% CI (0.610, 0.902)].

In the NAc core, significant main effects of lesion [*F*(1,16) = 69.673, *p* < 0.001, ηp^2^ = 0.813, 95% CI (0.591, 0.897)] and pre-exposure [*F*(1,16) = 13.981, *p* = 0.002, ηp^2^ = 0.466, 95% CI (0.096, 0.690)] were likewise accompanied by a significant lesion × pre-exposure interaction [*F*(1,16) = 76.121, *p* < 0.001, ηp^2^ = 0.826, 95% CI (0.617, 0.904); Figures 10D,F].

In sham animals, sucrose pre-exposure was associated with significantly greater c-Fos expression in the whole NAc [PE vs. NPE; *p* < 0.001, Hedges’ *g* = 3.09, 95% CI (1.21, 4.97)], NAc shell [PE vs. NPE; *p* < 0.05, *g* = 1.36, 95% CI (0.06, 2.66)], and NAc core [PE vs. NPE; *p* < 0.05, *g* = 2.00, 95% CI (0.51, 3.49)], consistent with enhanced accumbal recruitment under the PE condition.

In contrast, neonatal MD lesions reversed this pre-exposure-dependent pattern: PE-MD-lesionanimals exhibited markedly lower c-Fos expression than NPE-MD-lesion animals in the whole NAc [*p* < 0.001, *g* = 6.56, 95% CI (5.27, 15.85)], shell [*p* < 0.001, *g* = 5.63, 95% CI (2.65, 8.61)], and core [*p* < 0.001, *g* = 5.07, 95% CI (2.35, 7.79)]. PE-MD-lesion animals also exhibited substantially lower c-Fos expression than PE-Sham animals in the whole NAc [*p* < 0.001, *g* = 7.56, 95% CI (4.74, 14.38)], shell [*p* < 0.001, *g* = 7.35, 95% CI (5.16, 15.54)], and core [*p* < 0.001, *g* = 6.03, 95% CI (3.94, 12.12)]. By contrast, c-Fos expression did not differ significantly between NPE-Sham and NPE-MD-lesion animals in the whole NAc, shell, or core (all *p* > 0.05; Figures 10D,F).

Together, these findings indicate that neonatal MD lesions markedly altered the pre-exposure-dependent pattern of accumbal recruitment during CTA, with concordant effects in the shell and core, while leaving basal c-Fos expression unchanged.

### 3.4 Experiment 3. Haloperidol restores latent inhibition following neonatal MD lesions

#### 3.4.1 Haloperidol does not alter total liquid consumption during the CTA paradigm

To determine whether haloperidol (HAL) affected drinking behavior independently of conditioned taste aversion, total liquid consumption was analyzed throughout the CTA paradigm using a three-way ANOVA with lesion status, pre-exposure condition, and treatment as between-subject factors.

Baseline liquid consumption did not differ according to lesion status [*F*(1,32) = 0.101, *p* = 0.750, ηp^2^ = 0.003, 95% CI (0.000, 0.110)], pre-exposure condition [*F*(1,32) = 1.423, *p* = 0.240, ηp^2^ = 0.043, 95% CI (0.000, 0.202)], or HAL treatment [*F*(1,32) = 0.031, *p* = 0.860, ηp^2^ = 0.001, 95% CI (0.000, 0.079); Figure 11A]. Likewise, total liquid consumption remained comparable across all groups during both the conditioning session and the test day (all *p* > 0.05).

**Figure 11:**
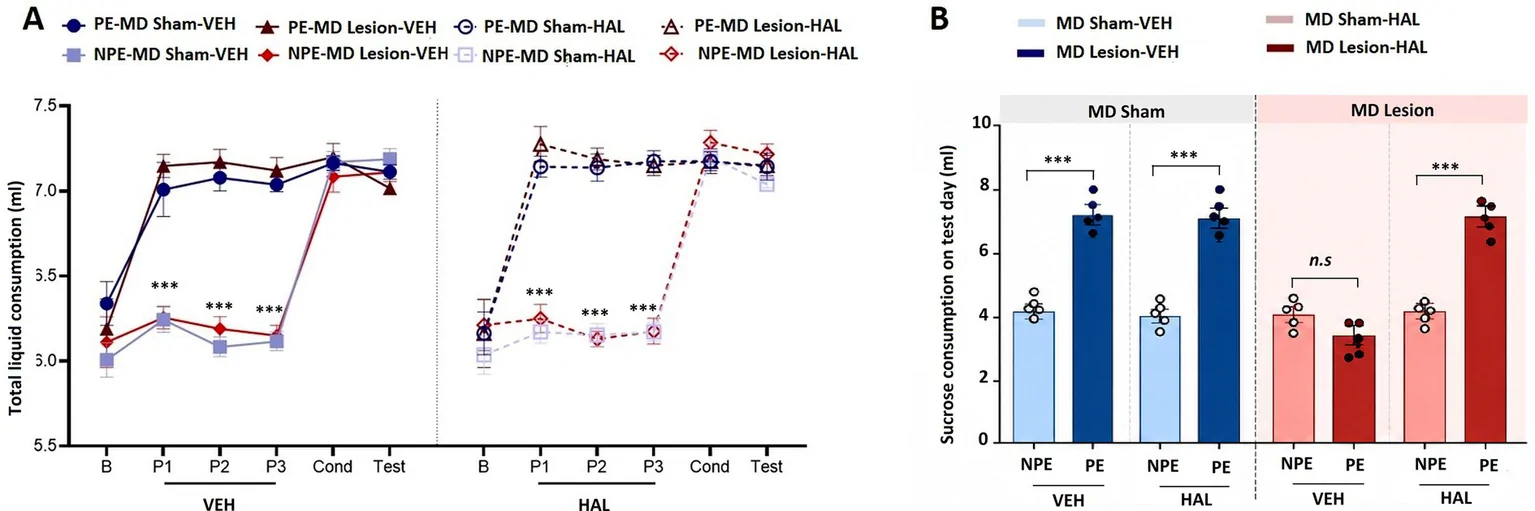
Haloperidol restored latent inhibition following neonatal MD lesions. **(A)** Total liquid consumption across baseline **(B)**, pre-exposure sessions (P1–P3), conditioning (Cond), and testing (Test) in vehicle- and haloperidol-treated groups. **(B)** Sucrose consumption during the test session. Data are presented as mean ± SEM (*n* = 5 per group). Statistical symbols denote the pairwise comparisons indicated in the figure. *n.s* > 0.05; ***p < 0.001.

During the three pre-exposure sessions, the three-way ANOVA consistently revealed a significant main effect of pre-exposure [P1: *F*(1,32) = 215.900, *p* < 0.001, ηp^2^ = 0.871, 95% CI (0.801, 0.911); P2: *F*(1,32) = 437.900, *p* < 0.001, ηp^2^ = 0.932, 95% CI (0.894, 0.952); P3: *F*(1,32) = 484.100, *p* < 0.001, ηp^2^ = 0.938, 95% CI (0.904, 0.956); Figure 11A], whereas neither lesion status nor HAL treatment significantly affected liquid consumption (all *p* > 0.05). Consistent with these findings, within-group analyses showed similar temporal patterns across all experimental groups: non-pre-exposed animals maintained baseline consumption during the pre-exposure phase and increased intake only during conditioning and testing, whereas pre-exposed animals exhibited increased consumption from the first pre-exposure session onward (all *p* < 0.001). These results indicate that HAL did not alter overall drinking behavior during any phase of the CTA paradigm.

#### 3.4.2 Haloperidol restores latent inhibition following neonatal MD lesions

Sucrose consumption on the test day was analyzed using a three-way ANOVA with lesion status, pre-exposure condition, and treatment as between-subject factors. Significant main effects were observed for lesion status [*F*(1,32) = 189.700, *p* < 0.001, ηp^2^ = 0.856, 95% CI (0.779, 0.900)], pre-exposure [*F*(1,32) = 982.200, *p* < 0.001, ηp^2^ = 0.968, 95% CI (0.950, 0.977)], and HAL treatment [*F*(1,32) = 197.800, *p* < 0.001, ηp^2^ = 0.861, 95% CI (0.786, 0.903)].

Significant interactions were also detected between pre-exposure and treatment [*F*(1,32) = 175.300, *p* < 0.001, ηp^2^ = 0.846, 95% CI (0.764, 0.893)], pre-exposure and lesion status [*F*(1,32) = 180.100, *p* < 0.001, ηp^2^ = 0.849, 95% CI (0.769, 0.895)], and treatment and lesion status [*F*(1,32) = 233.800, *p* < 0.001, ηp^2^ = 0.880, 95% CI (0.815, 0.916)]. Importantly, the pre-exposure × lesion × treatment interaction was significant [*F*(1,32) = 220.700, *p* < 0.001, ηp^2^ = 0.873, 95% CI (0.805, 0.911)], indicating that the effect of HAL depended on both lesion status and pre-exposure condition (Figure 11B).

Vehicle-treated sham animals showed a robust pre-exposure effect, with PE animals consuming substantially more sucrose than NPE animals [hedges’ *g* = 17.71, 95% CI (8.95, 26.47)]. In contrast, this pre-exposure-dependent behavioral pattern was disrupted in vehicle-treated MD-lesion animals: PE-MD-lesion rats consumed less sucrose than their NPE counterparts [hedges’ *g* = −1.41, 95% CI (−2.73, −0.07)] and markedly less than PE-sham animals.

The principal behavioral effect of HAL was observed in PE-MD-lesion animals. HAL markedly increased sucrose consumption in this group compared with vehicle-treated PE-MD-lesion animals [hedges’ *g* = 7.16, 95% CI (3.46, 10.86)]. Consequently, under HAL treatment, MD-lesion animals exhibited a robust pre-exposure effect, with PE animals consuming substantially more sucrose than NPE animals [hedges’ *g* = 15.15, 95% CI (7.63, 22.67)]. In contrast, HAL produced little change in sucrose consumption in NPE animals, either in sham [hedges’ *g* = 0.15, 95% CI (−0.96, 1.25)] or MD-lesion animals [hedges’ *g* = 0.45, 95% CI (−0.69, 1.57)]. Sucrose consumption in PE-sham animals was likewise not significantly altered by HAL compared with vehicle treatment (Figure 11B). Thus, the effect of HAL was primarily expressed in the PE-MD-lesion group rather than representing a generalized effect on sucrose consumption across experimental conditions.

Together, these findings indicate that the behavioral effect of systemic HAL was strongly dependent on lesion and pre-exposure status and was principally characterized by increased sucrose consumption in PE-MD-lesion animals, restoring the pre-exposure-dependent behavioral pattern that was disrupted following neonatal MD lesion. Because total fluid consumption was not significantly altered by HAL, this effect is unlikely to be explained solely by a nonspecific change in fluid intake.

#### 3.4.3 Haloperidol selectively restores prefrontal neuronal recruitment in pre-exposed MD-lesion animals

To determine whether HAL modified task-related neuronal recruitment following neonatal MD lesions, c-Fos expression was quantified in the DLO, PL, Cg, and M2 cortices.

In the DLO, three-way ANOVA revealed significant main effects of pre-exposure [*F*(1,32) = 217.200, *p* < 0.001, ηp^2^ = 0.872, 95% CI (0.803, 0.912)], HAL treatment [*F*(1,32) = 427.100, *p* < 0.001, ηp^2^ = 0.930, 95% CI (0.892, 0.951)], and MD lesion [*F*(1,32) = 285.076, *p* < 0.001, ηp^2^ = 0.899, 95% CI (0.844, 0.930)], together with significant two-way interactions and a significant pre-exposure × lesion × treatment interaction [*F*(1,32) = 424.563, *p* < 0.001, ηp^2^ = 0.930, 95% CI (0.891, 0.950); Figures 12A–D]. Vehicle-treated PE-MD-lesion animals exhibited markedly lower c-Fos expression than PE-Sham animals. Importantly, HAL treatment was associated with a pronounced increase in c-Fos expression in PE-MD-lesion animals, whereas no comparable HAL-associated change was observed in the other experimental conditions. Following HAL treatment, c-Fos expression in PE-MD-lesion animals approached that observed in PE-Sham animals. This similarity, however, should not be interpreted as evidence of complete functional normalization.

**Figure 12:**
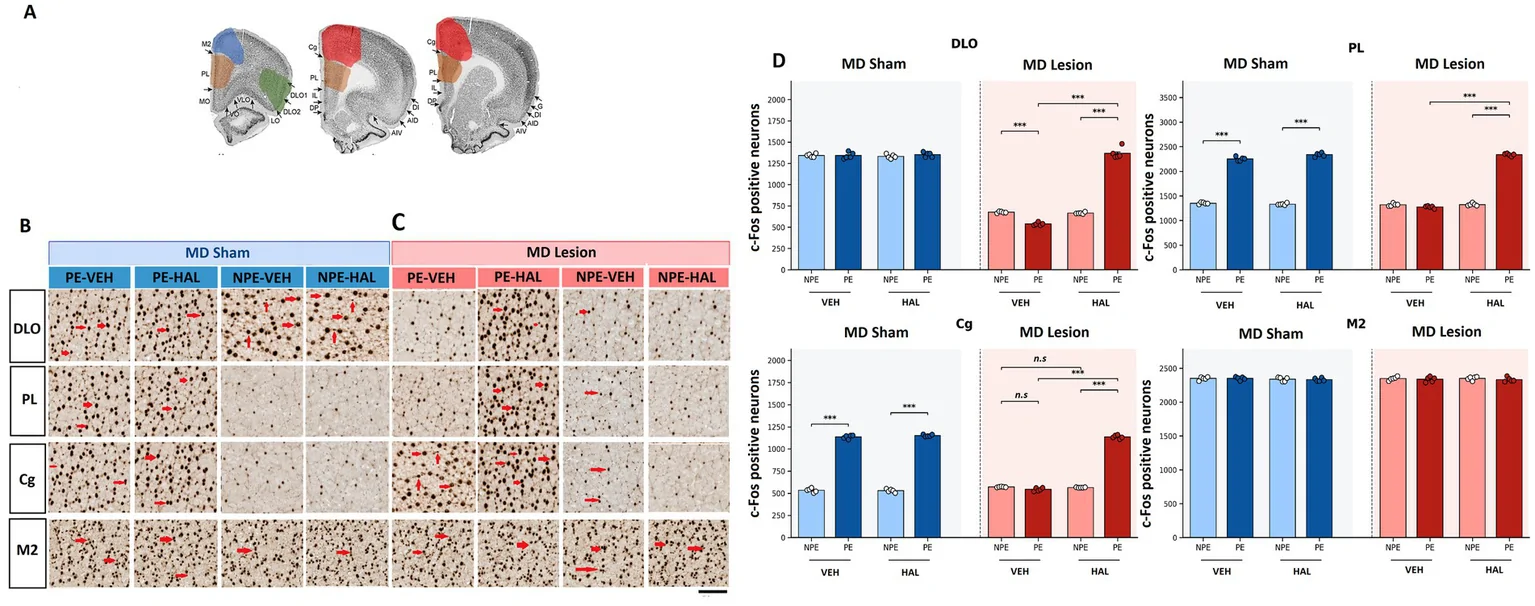
Haloperidol restored CTA-induced neuronal recruitment within prefrontal cortical regions of pre-exposed MD-lesion rats. **(A)** Coronal sections illustrating the prefrontal cortex (PFC) and secondary motor cortex (M2) regions sampled for c-Fos immunohistochemistry. **(B)** Representative c-Fos photomicrographs from MD-Sham groups in the DLO, PL, Cg, and M2. **(C)** Representative photomicrographs from MD-Lesion groups in the same regions; arrows indicate c-Fos-positive nuclei. **(D)** Density of c-Fos-positive nuclei in the DLO, PL, Cg, and M2. Scale bars: 40 μm. Data are presented as mean ± SEM (*n* = 5 per group) and were analyzed using three-way ANOVA (lesion × pre-exposure × treatment), followed by Holm-Šídák *post hoc* comparisons. *n.s* > 0.05; ***p < 0.001.

A similar treatment-dependent pattern was observed in the PL and Cg. Significant pre-exposure × lesion × treatment interactions were detected in both the PL [*F*(1,32) = 717.418, *p* < 0.001, ηp^2^ = 0.957, 95% CI (0.933, 0.970)] and Cg [*F*(1,32) = 789.277, *p* < 0.001, ηp^2^ = 0.961, 95% CI (0.939, 0.973); Figures 11A–C]. In the PL, HAL treatment was associated with a marked increase in c-Fos expression in PE-MD-lesion animals. Similarly, in the Cg, c-Fos expression increased following HAL treatment in PE-MD-lesion animals. In both regions, the values observed in HAL-treated PE-MD-lesion animals approached those of PE-Sham animals, whereas no comparable treatment-associated changes were observed in sham or NPE groups. Thus, the data indicate a strong HAL-associated increase in prefrontal c-Fos recruitment specifically under the PE-MD-lesion condition, rather than establishing complete normalization of prefrontal function.

In contrast, M2 showed no corresponding treatment-dependent pattern. Three-way ANOVA revealed no significant main effects of pre-exposure [*F*(1,32) = 0.955, *p* = 0.340, ηp^2^ = 0.029, 95% CI (0.000, 0.174)], HAL treatment [*F*(1,32) = 0.813, *p* = 0.370, ηp^2^ = 0.025, 95% CI (0.000, 0.165)], or lesion status [*F*(1,32) = 0.123, *p* = 0.728, ηp^2^ = 0.004, 95% CI (0.000, 0.114)]. Consistent with this result, c-Fos expression in PE-MD-lesion animals was similar under vehicle and HAL treatment (Figure 12D).

Together, these findings indicate that systemic HAL was associated with a marked increase in task-related c-Fos recruitment in the prefrontal regions examined specifically in PE-MD-lesion animals, whereas no corresponding effect was detected in the M2 control region. Although c-Fos values in HAL-treated PE-MD-lesion animals approached those observed in PE-Sham animals, these findings should not be interpreted as evidence of complete normalization or as demonstrating that changes in prefrontal c-Fos expression mediated the behavioral recovery.

#### 3.4.4 Haloperidol restores CTA-induced neuronal recruitment in the nucleus accumbens

To determine whether HAL modified task-related neuronal recruitment in the nucleus accumbens (NAc) following neonatal MD lesions, c-Fos expression was analyzed using a three-way ANOVA with lesion status, pre-exposure condition, and treatment as between-subject factors.

In the NAc, significant main effects were observed for pre-exposure [*F*(1,32) = 267.661, *p* < 0.001, ηp^2^ = 0.893, 95% CI (0.835, 0.926)], HAL treatment [*F*(1,32) = 257.912, *p* < 0.001, ηp^2^ = 0.890, 95% CI (0.830, 0.924)], and MD lesion [*F*(1,32) = 196.816, *p* < 0.001, ηp^2^ = 0.860, 95% CI (0.785, 0.903)]. Significant lesion × pre-exposure [*F*(1,32) = 188.278, *p* < 0.001, ηp^2^ = 0.855, 95% CI (0.777, 0.899)], lesion × treatment [*F*(1,32) = 178.283, *p* < 0.001, ηp^2^ = 0.848, 95% CI (0.767, 0.894)], and pre-exposure × treatment [*F*(1,32) = 280.110, *p* < 0.001, ηp^2^ = 0.897, 95% CI (0.841, 0.929)] interactions were also detected. Importantly, the lesion × pre-exposure × treatment interaction was significant [*F*(1,32) = 136.516, *p* < 0.001, ηp^2^ = 0.810, 95% CI (0.710, 0.868); Figures 13B,C].

**Figure 13:**
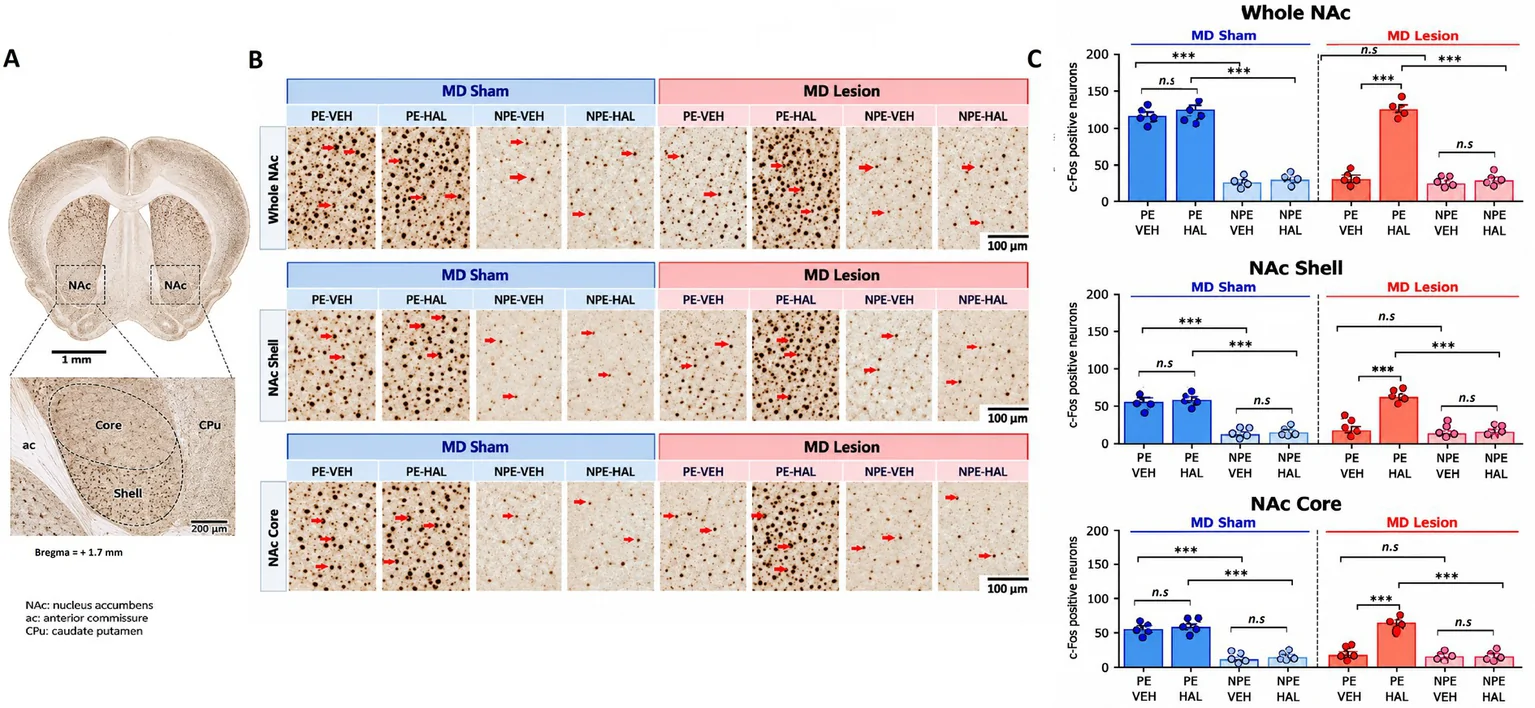
Haloperidol restored CTA-induced neuronal recruitment within the nucleus accumbens of pre-exposed MD-lesion rats. **(A)** Coronal sections showing the NAc shell and core regions used for quantification. **(B)** Representative photomicrographs of c-Fos immunolabeling in the NAc core and shell across vehicle- and haloperidol-treated NPE and PE groups; arrows indicate c-Fos-positive nuclei. **(C)** Density of c-Fos-positive nuclei in the whole NAc, shell, and core. Scale bars: 100 μm. Data are presented as mean ± SEM (*n* = 5 per group) and were analyzed using three-way ANOVA (lesion × pre-exposure × treatment), followed by Holm-Šídák *post hoc* comparisons. Statistical symbols denote the pairwise comparisons indicated in the figure: *n.s* > 0.05; ***p < 0.001.

Vehicle-treated PE-MD-lesion animals exhibited markedly lower c-Fos expression than PE-Sham animals. HAL treatment was associated with a pronounced increase in c-Fos expression in PE-MD-lesion animals, whereas no comparable HAL-associated increase was observed in the NPE-MD-lesion group. Thus, the HAL effect was strongly dependent on both lesion status and pre-exposure condition.

The NAc shell and core exhibited similar treatment-dependent patterns. In the NAc shell, significant main effects of lesion [*F*(1,32) = 196.777, *p* < 0.001, ηp^2^ = 0.860, 95% CI (0.785, 0.903)], pre-exposure [*F*(1,32) = 2038.158, *p* < 0.001, ηp^2^ = 0.985, 95% CI (0.976, 0.989)], and HAL treatment [*F*(1,32) = 247.839, *p* < 0.001, ηp^2^ = 0.886, 95% CI (0.824, 0.921)] were accompanied by a significant lesion × pre-exposure × treatment interaction [*F*(1,32) = 193.355, *p* < 0.001, ηp^2^ = 0.858, 95% CI (0.781, 0.901); Figures 13B,C]. HAL treatment was associated with a marked increase in c-Fos expression in PE-MD-lesion animals, whereas little change was observed in NPE-MD-lesion animals.

Similarly, in the NAc core, significant main effects of lesion [*F*(1,32) = 193.355, *p* < 0.001, ηp^2^ = 0.858, 95% CI (0.781, 0.901)], pre-exposure [*F*(1,32) = 2060.341, *p* < 0.001, ηp^2^ = 0.985, 95% CI (0.977, 0.989)], and HAL treatment [*F*(1,32) = 263.508, *p* < 0.001, ηp^2^ = 0.892, 95% CI (0.833, 0.925)] were detected, together with a significant lesion × pre-exposure × treatment interaction [*F*(1,32) = 196.777, *p* < 0.001, ηp^2^ = 0.860, 95% CI (0.785, 0.903); Figures 13B,C]. In PE-MD-lesion animals, c-Fos expression increased following HAL, whereas NPE-MD-lesion animals showed little corresponding change.

Together, these findings indicate that systemic HAL was associated with a marked increase in task-related c-Fos recruitment throughout the NAc specifically in PE-MD-lesion animals, paralleling the restoration of the pre-exposure-dependent behavioral effect.

## 4 Discussion

### 4.1 Developmental MD disruption is associated with long-lasting alterations across MD–PFC–NAc regions

The present study shows that neonatal lesions of the mediodorsal thalamus (MD) are associated with long-lasting molecular, regional, and behavioral alterations across MD–PFC–NAc regions. Four findings are particularly relevant. First, neonatal MD lesions disrupted the normal pre-exposure-dependent behavioral pattern in the conditioned taste aversion (CTA) paradigm. Second, they were associated with reduced basal c-Fos expression in the dorsolateral orbitofrontal (DLO), prelimbic (PL), and cingulate (Cg) cortices, while sparing the secondary motor cortex (M2), and with regionally heterogeneous changes in task-related c-Fos recruitment during CTA. Third, D2 receptor (D2R) expression was persistently increased in prefrontal regions at transcript, protein, and immunohistochemical levels, without a significant increase in the number of D2R-immunopositive neurons. Finally, systemic haloperidol restored the behavioral pre-exposure effect in MD-lesion animals and was accompanied by marked changes in c-Fos recruitment within PFC and NAc regions. These convergent findings support a developmental association between early MD disruption, persistent remodeling of prefrontal dopaminergic signaling, and altered patterns of c-Fos expression across anatomically interconnected thalamo-cortico-striatal regions, while not establishing a causal sequence among these changes.

The developmental timing of the lesion is important to this interpretation. Excitotoxic damage was induced at postnatal day 4, during a period of intense synaptogenesis, thalamocortical maturation, and activity-dependent refinement of cortical circuits (Cotman et al., 1984). Reciprocal MD–PFC projections are still developing at this stage (Van Eden, 1986), whereas corticostriatal pathways continue to mature during the following postnatal weeks (Chen et al., 2025). Disruption of MD input during this developmental period could therefore influence the maturation of distributed neural networks rather than produce effects restricted to the thalamus itself.

This interpretation is consistent with the anatomical organization of the MD–PFC–NAc circuit. The MD maintains extensive reciprocal connections with multiple prefrontal regions, which in turn project densely to the NAc, forming a network involved in executive function, behavioral flexibility, selective attention, and adaptive learning (Leonard, 1969; Krettek and Price, 1977; Groenewegen, 1988; Berendse and Groenewegen, 1990; Floresco et al., 2009). Our previous work demonstrated persistent reductions in spontaneous neuronal recruitment within the PFC and amygdala following neonatal MD lesions (Ouhaz et al., 2017). The present findings extend this observation by showing that early MD disruption is also associated with persistent D2R remodeling and altered task-related recruitment of prefrontal and accumbal regions during LI.

### 4.2 Developmental remodeling of prefrontal dopamine D2 receptors following neonatal MD lesions

A major finding of the present study is the persistent increase in prefrontal D2R expression after neonatal MD lesions. Importantly, this phenotype was supported by three complementary levels of analysis: increased D2R transcript, increased D2R protein, and increased immunohistochemical labeling in prefrontal regions. The absence of a corresponding effect in M2 argues against a uniform cortical change. Moreover, the number of D2R-immunopositive neurons was not significantly increased, whereas labeling density and labeling per D2R-positive neuron were higher in the DLO, PL, and Cg. Taken together, these observations are most consistent with increased D2R expression within D2R-positive prefrontal cells rather than an increase in the number of D2R-expressing cells.

The susceptibility of prefrontal D2Rs to developmental disruption is consistent with the prolonged maturation of the dopaminergic system. D2R expression increases progressively throughout juvenile development before reaching adult levels (Tarazi et al., 1998; Andersen et al., 2002; Brenhouse et al., 2008). During this same period, thalamocortical activity contributes to synaptic refinement, dendritic maturation, and stabilization of cortical microcircuits. Early postnatal MD lesions produce enduring reductions in prefrontal dendritic complexity and spine density (Marmolejo et al., 2012; Ouhaz et al., 2015), providing a plausible developmental context in which altered thalamic input could be accompanied by long-term reorganization of dopamine-sensitive cortical networks.

Developmental perturbations, including early social isolation, have also been reported to produce persistent changes in D2R expression within corticolimbic circuits (Djouma et al., 2006; Han et al., 2012).

The mechanisms underlying the D2R increase remain speculative. Loss of MD-derived excitatory input could interfere with activity-dependent developmental programs governing PFC maturation and dopamine receptor-dependent synaptic organization. Alternatively, increased D2R expression could represent a compensatory response to chronically reduced cortical recruitment, consistent with activity-dependent homeostatic mechanisms (Turrigiano et al., 1998). The present basal c-Fos data are compatible with this possibility because DLO, PL, and Cg all showed lower basal c-Fos expression after MD lesion, whereas M2 was preserved. These mechanisms are not mutually exclusive and cannot be distinguished by the present experiments.

An alternative, but not mutually exclusive, explanation is that increased D2R expression represents a compensatory or homeostatic response to persistent alterations in prefrontal network recruitment following developmental MD disruption, rather than a primary mechanism responsible for the behavioral phenotype. Consistent with this possibility, our previous work demonstrated that neonatal MD lesions produce enduring reductions in neuronal recruitment within prefrontal regions (Ouhaz et al., 2017). The reduced basal c-Fos expression observed in the DLO, PL, and Cg in the present study provides additional evidence that altered prefrontal recruitment persists into adulthood. Under conditions of chronically altered network activity, changes in receptor expression may represent an adaptive mechanism aimed at maintaining or rebalancing cellular and circuit function. Such an interpretation is consistent with the broader concept of homeostatic plasticity, whereby persistent changes in neuronal activity can induce compensatory adjustments in synaptic and receptor-related mechanisms to stabilize network function (Turrigiano et al., 1998).

From this perspective, the persistent increase in prefrontal D2R expression could represent a downstream consequence of developmental MD dysfunction and long-term cortical reorganization, rather than the initiating cause of the latent-inhibition deficit. Indeed, the present experiments cannot determine whether D2R upregulation precedes the behavioral alteration, contributes to its expression, or emerges as an adaptive response to persistent changes in prefrontal circuitry. The absence of a significant increase in the number of D2R-immunopositive neurons, together with increased D2R labeling within D2R-positive prefrontal cells, is compatible with altered receptor regulation within an existing cellular population rather than recruitment of a new D2R-expressing population. However, whether this molecular adaptation is functionally compensatory, maladaptive, or partly both remains unresolved.

The behavioral effect of haloperidol should also be considered within this framework. Although restoration of the pre-exposure-dependent behavioral pattern by D2R blockade indicates that the adult phenotype remains sensitive to D2-dependent modulation, it does not establish that increased prefrontal D2R expression caused the LI deficit. In particular, systemic haloperidol can act at D2Rs in the NAc and other dopaminergic regions in addition to the PFC. Thus, the present findings are compatible with a model in which early MD damage produces persistent circuit-level alterations, accompanied by secondary/homeostatic remodeling of prefrontal D2R expression, with D2-sensitive mechanisms subsequently modulating the expression of the behavioral phenotype. Longitudinal studies examining the emergence of D2R alterations across development, together with region-specific manipulation of prefrontal D2Rs, will be required to distinguish whether D2R upregulation is causally involved in the LI deficit or represents a compensatory consequence of developmental MD dysfunction.

It is also important to distinguish receptor expression from receptor function. Although the convergence of qRT-PCR, Western blotting, and immunohistochemistry provides strong evidence for persistent D2R upregulation, increased receptor abundance does not necessarily imply increased D2 signaling, altered receptor affinity, receptor occupancy, or increased endogenous dopaminergic tone. In addition, the present immunohistochemical approach does not resolve the cellular or subcellular localization of the increased D2R signal. Thus, the data cannot determine whether the relevant changes are predominantly postsynaptic, presynaptic, or restricted to specific neuronal populations. Cell-type-specific localization and direct measurements of dopamine release and D2R signaling will be required to resolve these possibilities.

D2R remodeling occurred in parallel with altered prefrontal recruitment during LI. This anatomical correspondence, together with the behavioral effect of D2 receptor blockade, makes altered dopaminergic signaling a plausible contributor to the phenotype. However, the present study does not establish that D2R upregulation causes the c-Fos changes or the LI deficit. This distinction is particularly important because haloperidol was administered systemically and therefore could act at D2 receptors in the PFC, NAc, and other dopaminergic targets. The pharmacological experiment consequently supports involvement of D2-sensitive mechanisms but cannot localize the relevant site of action to the PFC or demonstrate that the behavioral rescue results specifically from blockade of the upregulated prefrontal D2Rs. This interpretation is consistent with evidence that excessive D2R signaling can produce persistent abnormalities in prefrontal dopaminergic regulation and inhibitory transmission (Kellendonk et al., 2006; Li et al., 2011), and that haloperidol can restore disrupted LI in lesion-based neurodevelopmental models (Schmajuk et al., 2000; Ouhaz et al., 2014).

### 4.3 Regionally heterogeneous PFC and NAc recruitment is associated with disrupted latent inhibition

The c-Fos findings indicate that neonatal MD lesions are associated with altered patterns of c-Fos expression across PFC and NAc regions within the anatomically defined MD–PFC–NAc system, but they also reveal important regional heterogeneity. This heterogeneity is informative because the DLO, PL, and Cg did not show interchangeable response profiles. In the DLO, the lesion effect during CTA was strongly dependent on pre-exposure: c-Fos expression was markedly reduced in pre-exposed MD-lesion animals, whereas NPE sham and MD-lesion animals were similar. In the PL, MD lesions produced a large reduction under both PE and NPE conditions, but a smaller pre-exposure-dependent increase remained detectable within MD-lesion animals. In the Cg, by contrast, the lesion effect predominated, and there was no significant pre-exposure-dependent modulation. The data therefore support regionally differentiated consequences of developmental MD disruption rather than a unitary reduction in PFC recruitment.

The basal c-Fos findings further qualify the interpretation of the CTA data. DLO, PL, and Cg already exhibited markedly lower c-Fos expression in MD-lesionanimals under basal conditions, whereas M2 did not. Task-related reductions in these prefrontal regions therefore occur on a background of altered basal cortical recruitment and should not automatically be interpreted as LI-specific deficits. Rather, the CTA experiment indicates that pre-exposure and lesion status interact differently across prefrontal subregions superimposed on this persistent basal alteration. This distinction is important when relating c-Fos expression to behavior.

A related pattern was observed in the NAc. Under basal conditions, c-Fos expression was similar between sham and MD-lesion animals in the whole NAc and in its shell and core subdivisions. During CTA, however, the normal pre-exposure-associated recruitment pattern was disrupted by neonatal MD lesions, with pronounced reductions in pre-exposed MD-lesionanimals. The parallel effects in shell and core suggest that the impact on accumbal recruitment is not restricted to a single subdivision. Nevertheless, the present design does not establish the functional contribution of either subdivision to the behavioral phenotype.

The behavioral data should likewise be interpreted as a disruption of the normal pre-exposure-dependent pattern rather than simply as an absence of a statistically significant PE–NPE difference. In sham animals, pre-exposure produced the expected increase in sucrose consumption on the test day. In MD-lesion animals, this relationship was lost and, in the haloperidol experiment under vehicle treatment, the direction of the PE–NPE difference was abnormal. This pattern is compatible with disrupted LI, but the term “abolished” may obscure the fact that the lesion can produce an altered or reversed pre-exposure effect rather than merely eliminating group differentiation.

Importantly, neonatal MD lesions did not produce a generalized reduction in total liquid consumption across the CTA paradigm. This argues against nonspecific hypodipsia as an explanation for the sucrose-consumption phenotype. It does not, however, exclude differences in locomotor activity, motivational state, stress responsivity, or other behavioral processes that were not directly measured. These possibilities should therefore remain separate from conclusions supported by the consumption data.

The regional dissociations are compatible with functional heterogeneity among prefrontal territories. The DLO has been implicated in updating stimulus value and outcome expectancy, whereas the PL contributes to executive control and attentional allocation during associative learning (Dias et al., 1996, 1997; Birrell and Brown, 2000; Brown and Bowman, 2002). The present data are consistent with differential engagement of these regions, but c-Fos is an indirect and temporally integrated marker of neuronal recruitment. Accordingly, the observed c-Fos patterns cannot establish that any individual PFC or NAc region mediates the MD-lesion effect on CTA behavior, nor do they demonstrate impaired functional communication between regions. More broadly, these findings are consistent with the view that the MD participates in coordinating distributed cortical activity rather than acting solely as a passive relay (Parnaudeau et al., 2013, 2015; Mitchell et al., 2014; Wolff and Vann, 2019).

### 4.4 Haloperidol restores the behavioral pre-exposure effect and modifies regional PFC and NAc c-Fos expression

In the present study, we examined the effects of a single systemic injection of haloperidol (HAL) administered specifically during the pre-exposure phase of latent inhibition (LI) in a conditioned taste aversion (CTA) paradigm. The selected dose (0.1 mg/kg) was based on previous work demonstrating its efficacy in modulating LI without inducing nonspecific behavioral effects, including locomotor or social alterations (Sams-Dodd, 1999; Compton et al., 2001; Ouhaz et al., 2014). Importantly, this dose has previously been shown to facilitate LI in models of neonatal hippocampal dysfunction (Ouhaz et al., 2014).

Systemic haloperidol produced a striking condition-dependent behavioral effect. In MD-lesion animals, its principal behavioral action was a marked increase in sucrose consumption in the pre-exposed group, whereas consumption in the non-pre-exposed group changed little. Haloperidol therefore restored a robust pre-exposure-dependent behavioral pattern rather than producing a uniform change in sucrose consumption. This distinction is important because it argues against interpreting the treatment simply as a nonspecific shift in consummatory behavior.

The behavioral change was accompanied by marked increases in c-Fos expression in pre-exposed MD-lesion animals within DLO, PL, Cg, and NAc regions. In several regions, the resulting values approached those observed in pre-exposed sham animals. Nevertheless, similarity of group means should not be interpreted as proof of complete physiological normalization. c-Fos provides only an indirect index of recruitment, and the correspondence between behavioral recovery and c-Fos changes does not establish that the latter mediated the former.

Our observations are consistent with previous studies showing that D2 receptor antagonists facilitate LI and reverse attentional deficits induced by psychostimulants and other neurodevelopmental manipulations (Feldon and Weiner, 1991; Moser et al., 2000; Arad and Weiner, 2009; Ouhaz et al., 2014). The present findings extend these observations to developmental disruption of the MD. However, because haloperidol was administered systemically, the experiment cannot determine whether its effects arose from blockade of prefrontal D2Rs, accumbal D2Rs, other dopaminergic targets, or a combination of distributed actions. The pharmacological findings should therefore be interpreted as evidence that the adult phenotype remains sensitive to D2 receptor modulation, rather than as a region-specific rescue mechanism.

The mechanisms underlying this behavioral restoration remain to be elucidated. D2 receptors regulate cortical excitability, corticostriatal transmission, and synaptic plasticity, all of which may contribute to attentional control and adaptive learning (Seamans and Yang, 2004; Tritsch and Sabatini, 2012). D2 receptor blockade may therefore modify network dynamics in a manner that permits expression of the pre-exposure effect in MD-lesion animals. Direct tests of this hypothesis will require local pharmacological manipulations or region- and cell-type-specific D2R approaches, ideally combined with temporally resolved measurements of dopamine signaling and neural activity during pre-exposure, conditioning, and testing.

### 4.5 Limitations and future directions

Several limitations should be considered when interpreting the present findings. The relatively small sample size may limit the precision and generalizability of the estimated effects, despite the use of individual animals as biological replicates and the prospective determination of sample size. Replication in larger independent cohorts will therefore be important to establish the robustness of the observed molecular and behavioral effects.

The interpretation of the c-Fos findings also requires caution. c-Fos immunoreactivity is an indirect and temporally integrated marker of neuronal recruitment rather than a direct measure of neuronal firing, synaptic transmission, or functional connectivity. Consequently, the regional changes observed in the PFC and NAc identify patterns of recruitment associated with lesion status, pre-exposure, and pharmacological treatment, but do not demonstrate altered functional communication within the MD–PFC–NAc pathway. Establishing such interactions will require pathway-specific approaches combined with temporally resolved electrophysiological or calcium-based measurements.

Similar considerations apply to the interpretation of the dopaminergic findings. Although the convergence of qRT-PCR, Western blotting, and immunohistochemistry provides evidence for persistent changes in prefrontal D2R expression, these approaches do not directly assess receptor signaling efficacy, receptor occupancy, or the cellular and subcellular localization of the altered D2R signal. Moreover, dopamine release and extracellular dopamine levels were not directly measured. Increased D2R expression therefore cannot be equated with enhanced D2R signaling or altered dopaminergic tone. These limitations are particularly relevant to the haloperidol experiment. Because haloperidol was administered systemically, its behavioral effects cannot be anatomically localized to the PFC and may reflect D2R blockade in the NAc or other dopaminergic regions, either independently or in combination with prefrontal effects. Thus, restoration of the pre-exposure-dependent behavioral pattern demonstrates that the adult phenotype remains sensitive to D2-dependent modulation, but does not establish that blockade of the upregulated prefrontal D2Rs specifically mediates the behavioral rescue. More broadly, the relationships among D2R upregulation, altered regional c-Fos recruitment, and impaired LI remain associative, and the present experiments do not establish a causal sequence linking these endpoints.

An additional consideration concerns the nature of the neonatal MD lesion model. The lesion was induced during an early developmental period in which thalamocortical and corticostriatal circuits are undergoing substantial maturation, making this approach useful for investigating the long-term consequences of early disruption of MD input. However, the excitotoxic manipulation also produces permanent neuronal loss within the MD. The adult molecular, regional, and behavioral phenotypes observed here may therefore reflect both developmental reorganization resulting from early disruption of thalamic input and the persistent consequences of reduced MD neuronal integrity in adulthood. The present design cannot dissociate these components. Transient or reversible manipulation of MD function during defined developmental windows would help distinguish the consequences of developmental circuit disruption from those attributable to permanent neuronal loss. For the same reason, this experimental lesion should not be considered to reproduce the complex and multifactorial pathophysiology of schizophrenia or other neurodevelopmental disorders; rather, it provides a model for investigating specific developmental circuit mechanisms potentially relevant to these conditions.

Finally, the present study was conducted exclusively in male rats, and the findings therefore cannot be assumed to generalize to females. Potential sex-related differences in the development and regulation of dopaminergic and corticostriatal systems may influence both the consequences of neonatal MD disruption and the response to D2R modulation. Future studies incorporating both sexes will be required to determine whether the persistent D2R changes, alterations in LI and regional c-Fos recruitment, and sensitivity to haloperidol are sex dependent.

Together, these limitations define several priorities for future investigation. Replication in larger cohorts and inclusion of both sexes will help establish the robustness and generalizability of the findings. An important additional question concerns the developmental trajectory of the phenotype. We are currently initiating a follow-up study designed to characterize the temporal emergence of MD lesion-related cognitive, affective, structural, and functional alterations across successive developmental stages, including adolescence and adulthood. This approach should help determine when the different components of the phenotype first emerge and whether molecular and regional alterations precede, accompany, or follow the development of cognitive and affective abnormalities. Region-specific or cell-type-specific manipulation of D2Rs within the PFC and NAc could determine whether particular D2R populations contribute causally to the behavioral phenotype and its pharmacological modulation. Direct measurements of dopamine release and D2R signaling, combined with pathway-specific manipulations and temporally resolved electrophysiological or calcium-based recordings, would further clarify how developmental MD disruption alters dopaminergic regulation and regional network dynamics during pre-exposure, conditioning, and testing. Such approaches will be necessary to move from the associations identified here toward a mechanistic understanding of the long-term consequences of developmental MD dysfunction.

### 4.6 General conclusion

The present study provides evidence that an early excitotoxic lesion of the mediodorsal thalamus is followed by persistent alterations in prefrontal D2R expression, basal prefrontal c-Fos expression, task-related PFC and NAc recruitment, and pre-exposure-dependent CTA behavior. The regional c-Fos profiles were heterogeneous rather than uniform, indicating that developmental MD disruption has differentiated consequences across the DLO, PL, Cg, and NAc. Systemic haloperidol restored the behavioral pre-exposure effect and was accompanied by marked changes in regional c-Fos recruitment, demonstrating that important components of the adult phenotype remain pharmacologically modifiable.

Taken together, the findings support a model in which neonatal MD disruption is associated with persistent remodeling of prefrontal dopaminergic signaling and altered regional recruitment patterns across anatomically interconnected thalamo-cortico-striatal structures. The data do not establish that D2R upregulation causes the recruitment abnormalities or that c-Fos changes mediate the behavioral phenotype. Rather, they identify convergent molecular, regional, and behavioral consequences of early MD disruption that can now be tested with region-specific and temporally resolved approaches. In this sense, the model provides a tractable framework for investigating developmental circuit mechanisms relevant to cognitive phenotypes associated with thalamocortical and dopaminergic dysfunction, including those implicated in neurodevelopmental disorders such as schizophrenia, without implying that the lesion model reproduces the disorder itself.

## Data Availability

The original contributions presented in the study are included in the article/supplementary material, further inquiries can be directed to the corresponding author/s.
